# Metrological Evaluation of Dimensional and Surface Roughness of Thermoplastic PLA Parts in High-Speed MEX 3D Printing Using a Dodecahedron Benchmark Geometry

**DOI:** 10.3390/ma19153255

**Published:** 2026-08-01

**Authors:** Anna Bazan, Paweł Turek, Paweł Kubik

**Affiliations:** Department of Manufacturing Techniques and Automation, Rzeszów University of Technology, 35-959 Rzeszów, Poland; abazan@prz.edu.pl (A.B.); p.kubik@prz.edu.pl (P.K.)

**Keywords:** high-speed material extrusion, polymer material, poly(lactic acid) (PLA), manufacturing consistency, complex symmetry geometry, dimensional-geometric accuracy, surface roughness, 3D printing strategies

## Abstract

This study addresses the influence of process conditions on the dimensional accuracy, geometric deviations, and surface quality of PLA parts manufactured using high-dynamics material extrusion (MEX) technology. The aim was to identify the dominant sources of variability and to assess within-condition manufacturing consistency and inter-machine consistency. The investigation considered two 3D printers, nine build locations on the working platform, two printing strategies (layer-by-layer and model-by-model), and model face orientation. Additionally, an exploratory comparison of aligned and random seam configurations and an analysis of local temperature variations within the build chamber were performed. Regular dodecahedron geometries were manufactured using a Bambu Lab P1S system and processed under identical high-quality printing parameters. Dimensional measurements were performed using a Linear 100 universal length measuring machine, while full-field geometric deviations were acquired using a GOM Scan 1 structured-light 3D scanner. Surface roughness (Ra) was measured with a MarSurf XR 20 profilometer. Part orientation is the dominant source of dimensional variability, representing the largest relative contribution to linear deviation in the mixed-effects model (ΔR^2^ = 0.776), while local temperature variations near the printing zone were associated with location-dependent dimensional deviations. Within the supplementary temperature dataset, the regression model including temperature and printers explained 66% of the variability in mean linear dimension. This association provides indirect evidence of a thermal contribution but does not establish direct causality. The layer-by-layer strategy provided better dimensional stability than the model-by-model approach. In the exploratory seam comparison, seam configuration did not explain the orientation-dependent LD pattern. Surface roughness variability was primarily geometry-driven (ΔR^2^ = 0.852). Variability between independent manufacturing series and specimens for linear deviation and Ra was low after accounting for the investigated factors, indicating consistent process performance under constant settings; however, the present design did not allow measurement repeatability and reproducibility to be separated. In conclusion, dimensional accuracy in high-dynamics MEX is strongly associated with part orientation, while thermal variations may represent an additional contributing factor; however, the observed correlation between thermal conditions and dimensional variability does not establish direct causality.

## 1. Introduction

Contemporary additive manufacturing technologies, particularly thermoplastic material extrusion (MEX), are undergoing an intensive transformation toward high-dynamics linear motion systems [1,2]. The widespread adoption of modern kinematic architectures, such as CoreXY systems, has enabled feed rates and accelerations that vastly exceed conventional standards [3]. This results in a radical reduction in production cycles, a phenomenon highly desirable from an industrial implementation perspective [1,2,3]. Nevertheless, the pursuit of maximizing volumetric throughput entails [4] a trade-off between manufacturing time [3], geometric fidelity, and part surface quality [5,6,7]. In this context, a rigorous metrological evaluation of the process capability of these machines becomes a key challenge, with particular emphasis on the within-condition manufacturing consistency; inter-machine consistency of producing models from popular polymers such as poly(lactic acid) (PLA).

In the accuracy analysis of high-speed systems, most attention has historically been paid to positioning errors resulting from inertial forces and frame natural vibrations [8,9]. However, the development of advanced vibration compensation algorithms effectively minimizes these undesirable mechanical phenomena [10,11]. Assuming that structural rigidity and kinematic calibration constitute an optimized, constant process background, the dominant source of dimensional deviations and surface roughness fluctuations shifts to non-mechanical factors [12,13]. These primarily include data preparation software parameters [14], rheological phenomena related to polymer flow dynamics [15,16], and spatial-thermal conditions inside the build chamber [17]. The varying location of the object on the platform exposes it to asymmetric effects of cooling systems and a non-uniform distribution of temperature gradients, which directly determines processing shrinkage and dimensional stability along the *X*, *Y*, and *Z* axes [18,19].

Particularly for semi-crystalline polymers like poly(lactic acid) (PLA), the thermal history during the 3D printing process governs its crystallization kinetics, which in turn directly impacts the volumetric shrinkage and final dimensional fidelity of the printed parts [20]. The inherent low crystallinity and relatively slow crystallization rate of PLA, combined with the rapid and uneven cooling typical of high-speed MEX processes, lead to internal thermal stresses, causing significant thermal-induced volume shrinkage and potential warpage [21]. Furthermore, the internal structure of the printed part, specifically the choice of infill density and pattern, plays a critical role in mitigating these dimensional variations while also dictating the mechanical characteristics of the component [22]. Understanding these foundational material and algorithmic factors is crucial for evaluating the true boundaries of dimensional manufacturing condidtency in high-dynamics manufacturing.

An objective assessment of the impact of the aforementioned variables on model quality requires reference to standardized diagnostic procedures [23]. In additive manufacturing metrology, models defined in the ISO/ASTM 52902 standard [23] are a widely recognized foundation, encompassing geometrized objects for assessing resolution, hole and pin accuracy, and surface roughness (Ra, Rz parameters) [24,25,26]. In engineering practice, thin-walled structures and stepped artifacts are also conventionally used to facilitate the isolation of errors along the machine’s primary axes [13].

Despite the existence of these normative standards, the dynamic advancement of high-speed systems reveals significant gaps in conventional testing methods. Classical models defined in standards, designed with significantly lower 3D printing speeds in mind, often fail to fully isolate errors resulting from abrupt acceleration vector changes [27] or non-linear extrusion delays [28], which compound in corner zones and during sudden axis reversals [14]. Consequently, the scientific community is seeking new, complementary test geometry solutions. These include the utilization of complex symmetry bodies printed serially to map deviations across multiple axes simultaneously, as well as specialized artifacts featuring non-linear toolpaths [14]. They enable a more effective verification of multi-axis interpolation errors and the identification of surface defects caused by material volumetric flow fluctuations [21,28] and non-uniform cooling under directional blowing [29].

In response to the outlined methodological gap, the objective of this study is to conduct a comprehensive analysis of the dimensional-geometric accuracy and surface texture of PLA models fabricated via high-dynamics MEX technology. Bearing in mind the diagnostic limitations of traditional normative objects, this research implements serially manufactured specimens featuring complex regular dodecahedron geometry. The experiment focuses on the isolation and quantification of errors arising from thermophysical and algorithmic factors, bypassing the need to replicate classical standardized tests. This approach will enable a verification of the practical boundaries of within-condition manufacturing consistency and inter-machine consistency of the investigated technology, thereby providing critical guidelines for process parameter optimization in modern, high-performance 3D printing.

## 2. Materials and Methods

**Figure 1:**
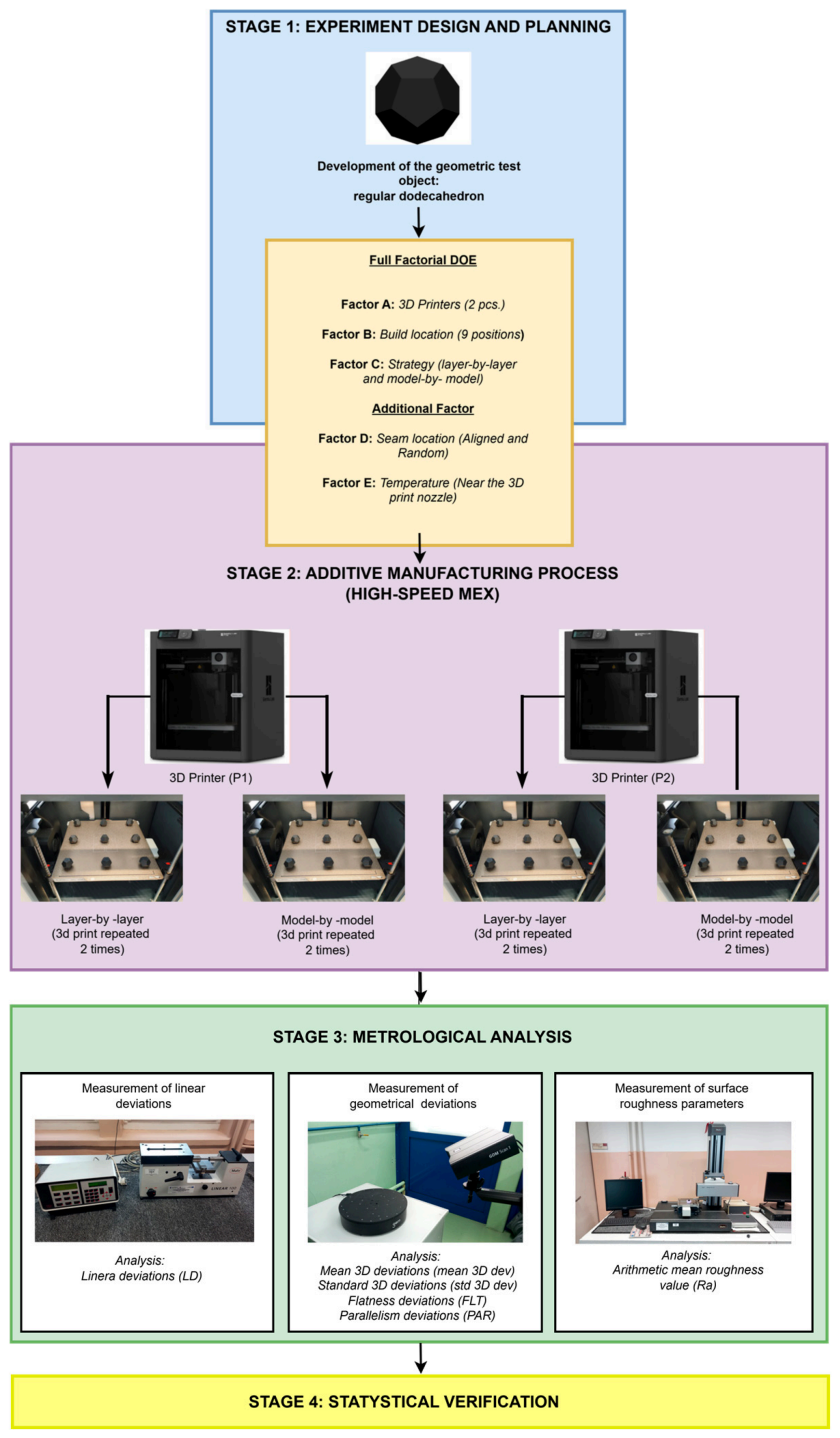
General scheme of the adopted research procedure.

The main objective of the conducted research was a comprehensive evaluation of within-condition manufacturing consistency and inter-machine consistency of the dimensional-geometric accuracy and surface texture of models fabricated using high-dynamics MEX technology (Figure 1). Bearing in mind the diagnostic limitations of traditional, standardized test objects, a model with complex symmetry in the shape of a regular dodecahedron was selected for the experiment. 

**Figure 2:**
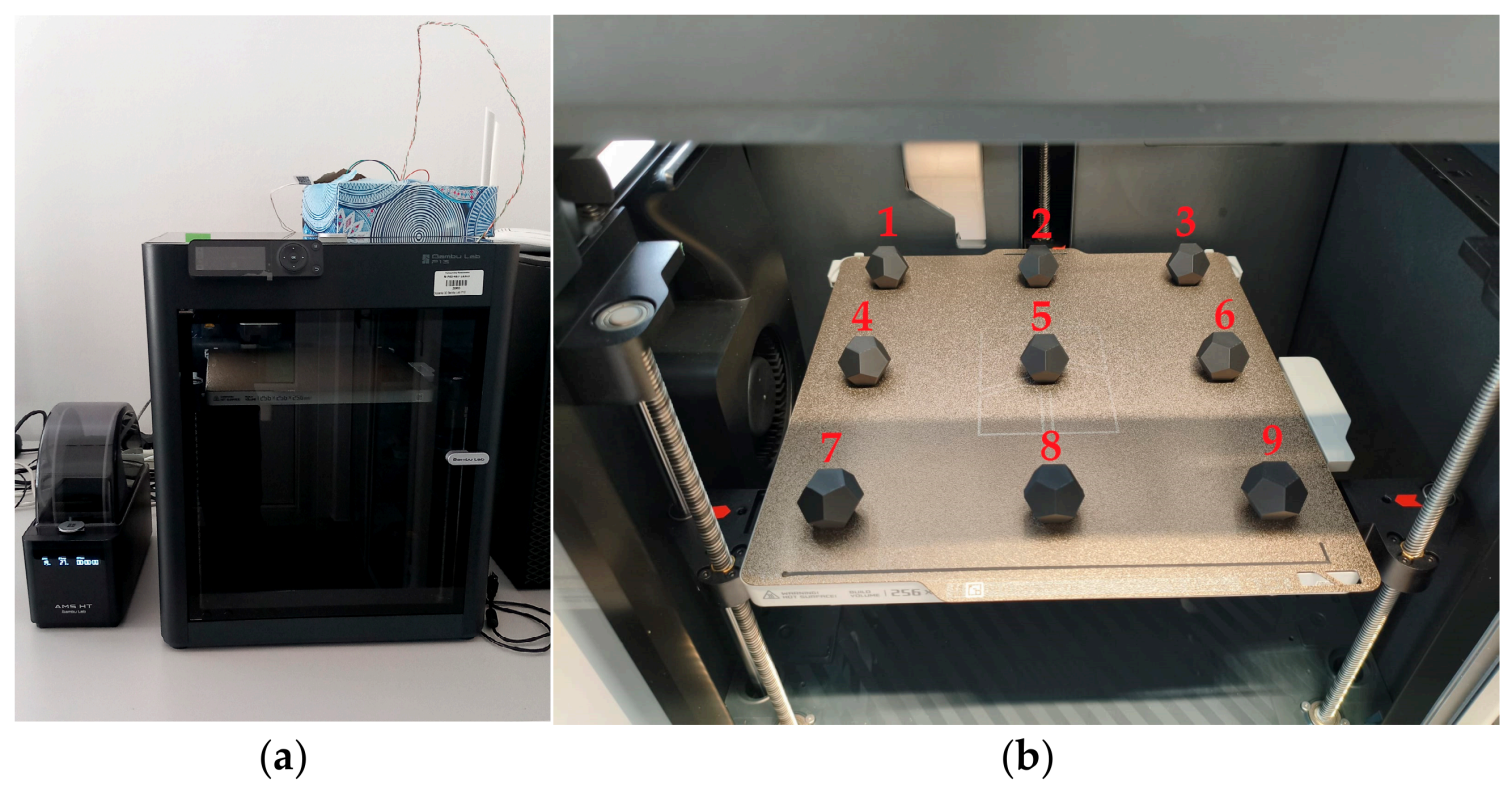
Model Fabrication Process: (**a**) Bambu Lab P1S Printer; (**b**) Arrangement of Fabricated Models within the Printer Workspace (9 build locations).

A detailed summary of the experimental design, including the number of manufacturing series, specimens, build positions, printing strategies, seam configurations, and measurements performed, is provided in Table 1. A manufacturing series was defined as one complete print job comprising nine specimens, one at each predefined build position. The specimens were manufactured in the following order: 3, 2, 6, 5, 1, 4, 9, 8, and 7 (Figure 2b). Two independent manufacturing series were produced for each printer–strategy combination in the main experiment. Before each manufacturing series, the printer was calibrated. Build positions were fixed throughout the study to preserve their spatial reference within the build platform. In the model-by-model strategy, the manufacturing sequence was identical for all print series and did not follow the numerical order of the build-position labels; therefore, the effects of build position and manufacturing order cannot be completely disentangled.

### 2.1. Development and Fabrication of the Regular Dodecahedron Model

The geometry of the test object, in the form of a regular dodecahedron, was designed in a CAD environment using Siemens NX software (version 2306). Upon completion of the modeling process, the three-dimensional solid was exported to the Stereolitography (STL) file. In order to eliminate the faceting error during the conversion of the model into a triangle mesh, increased export accuracy parameters were applied. An angular tolerance of 1° and a chordal tolerance of 0.005 mm were defined, which allowed for high-fidelity reproduction of the nominal geometry. Subsequently, the prepared STL file was imported into the Bambu Studio (version 1.9.1) software. Within this environment, the technological process preparation was performed, enabling the generation of the control code necessary to manufacture the models using the Bambu Lab P1S 3D printer (operating on firmware version 01.05.02.00) (Bambu Lab, Shenzhen, China) (Figure 2a). To evaluate the repeatability and reproducibility of the dimensional-geometric accuracy and surface roughness under demanding conditions, all models were produced using the predefined 0.08 mm High Quality print profile on a machine equipped with a stock 0.4 mm stainless steel nozzle (Figure 2b). To achieve maximum model density, the default settings were modified to a 100% solid infill density with a rectilinear pattern, maintaining a vertical resolution layer height of 0.08 mm (with an initial layer height of 0.12 mm). 

To isolate the structural and thermal effects of toolpath planning and layer sequence configurations, all kinematic parameters and velocity profiles linked to the 0.08 mm High Quality profile were kept strictly constant throughout the entire investigation. The manufacturing process was systematically varied across only two primary experimental factors:

- Seam Position: The placement of the layer-start acceleration points was alternated between: **#**1: Aligned (where the slicer attempts to hide the seam along internal corners) and **#**2: Random (where start points are distributed stochastically across the perimeter to analyze its impact on geometric deviations and surface roughness);
- Print Sequence Mode: The configuration of the manufacturing sequence on the build plate was varied between: **#**1: Layer-by-layer: All models on the platform are built simultaneously, advancing layer by layer, which introduces a significant cooling and travel time between individual layers of a single model **#**2: Model-by-model: Each specimen is fully completed individually before the print head moves to the next designated position on the build plate, minimizing the intra-layer cooling interval and isolating thermal gradients;

The material utilized for the fabrication of all experimental specimens was commercial Bambu Lab PLA Matte filament (Charcoal Gray, Batch No. 20231108A, from a single 1 kg spool to eliminate material variability). To prevent any moisture-induced degradation, boiling defects, or volumetric flow fluctuations during high-speed extrusion, the material underwent a rigorous conditioning procedure. Prior to the 3D printing process, the filament was thoroughly dried in a convective filament dryer at 55 °C for 6 h prior to the print jobs and maintained during the process in an Automated Material System (AMS) with fresh silica gel desiccant, keeping the internal relative humidity (RH) strictly below 15%. The nozzle temperature was set to 220 °C, and the heated bed temperature was maintained at 35 °C. Flow parameters were finely calibrated, with the extrusion flow ratio set to 0.98 and the Flow Dynamics (Pressure Advance) compensation set to a K-factor of 0.022. Prior to the print run, the machine underwent automated calibration routines, including automatic bed leveling (ABL) and active vibration-compensation (Input Shaping) frequency sweeps for the X and Y axes using the printer’s built-in internal accelerometers. To fully characterize the high-speed Material Extrusion (MEX) process and justify its classification, a comprehensive set of kinematic and dynamic parameters must be defined. While the outer-wall speed was intentionally constrained to 60 mm/s to ensure optimal surface finish and external dimensional fidelity within the 0.08 mm “High Quality” profile, the overall process kinematics were governed by significantly higher dynamic limits characteristic of high-speed systems. Specifically, the inner wall and infill printing speeds were set to 120 mm/s and 150 mm/s, respectively, supported by a high maximum volumetric flow rate limit of 21 mm^3^/s for the PLA Matte filament. Furthermore, the machine’s dynamic limits were defined by maximum acceleration values reaching 10,000 mm/s^2^ for both travel and internal extrusion moves. To mitigate ringing, ghosting, and positional errors induced by high inertial forces at these elevated accelerations, active vibration-compensation algorithms (Input Shaping) were enabled in the printer’s firmware. 

For the 0.08 mm High Quality profile, the actual average production time of a single dodecahedron model was approximately 32 min for the layer-by-layer strategy and about 28 min for the model-by-model strategy. The average effective printing speed (accounting for both working and travel moves) was approximately 115 mm/s. The actual volumetric flow rate during infill extrusion (100% rectilinear, path width 0.45 mm, layer height 0.08 mm at a speed of 150 mm/s) reached approximately 5.4 mm^3^/s, which remains safely below the declared machine limit of 21 mm^3^/s. Controller signal monitoring and onboard accelerometer data confirmed that thanks to the active Input Shaping algorithm, the programmed high speeds and accelerations (10,000 mm/s^2^) were dynamically executed within motion control tolerances, although a temporary speed reduction occurred in corner zones due to the kinematic limitations of the CoreXY mechanism.

The integration of high volumetric throughput, aggressive acceleration profiles, and active resonance compensation fundamentally distinguishes this setup from conventional MEX, thereby fully justifying its classification as a high-speed MEX process. The comprehensive manufacturing configuration and constant kinematic parameters extracted from the slicing system are summarized in Table 2.

**Figure 3:**
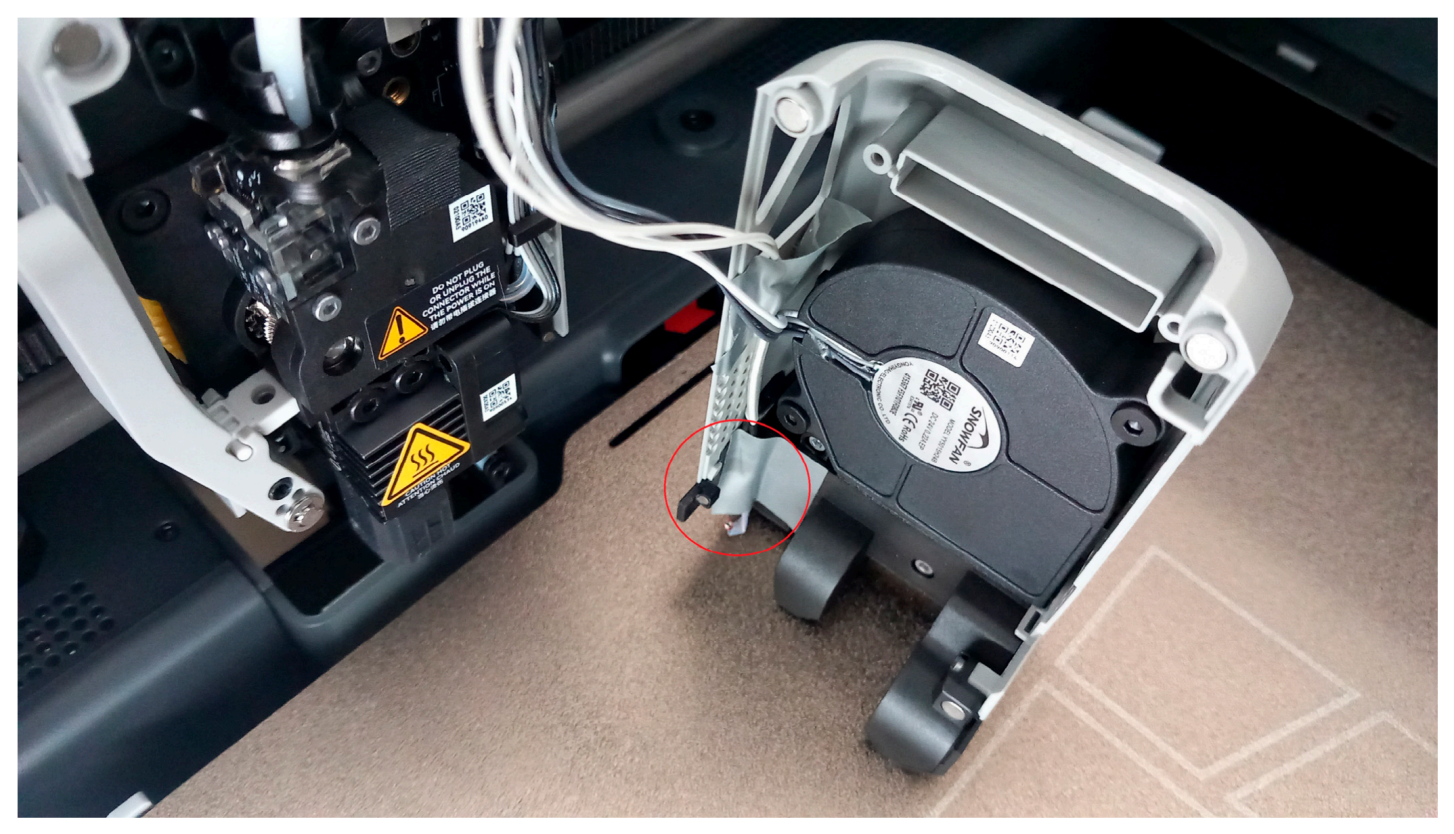
Experimental setup with auxiliary NTC thermistor (in the red circle) for local temperature monitoring.

During the 3D printing process, the local temperature in the vicinity of the print nozzle was recorded. For this purpose, a glass-encapsulated NTC thermistor (Dongguan Shinein Electronics Technology, Dongguan, China) with a nominal resistance of approximately 100 kΩ at room temperature was employed. The sensor was mounted on the print head at the location illustrated in Figure 3. The selection of the NTC thermistor was motivated by its high sensitivity within the range of a dozen to several dozen degrees Celsius, its compact dimensions, and its straightforward integration into the measurement system.

The NTC thermistor operated as the lower arm of a voltage divider configuration equipped with a 46.58 kΩ pull-up resistor. The voltage across the divider and the divider supply voltage were measured using an external ADS1115 analog-to-digital converter (Texas Instruments, Dallas, TX, USA) connected to a development board featuring an ESP32-WROOM-32D microcontroller (Espressif Systems, Shanghai, China). The thermistor resistance was determined via a ratiometric method based on the ratio of the divider voltage to its supply voltage. This approach effectively minimizes the influence of supply voltage fluctuations on the measurement accuracy. To reduce the impact of noise and interference, the ADS1115 was configured to operate at 860 samples per second; each individual reading was computed as the median of 64 samples for the node (thermistor) voltage and 16 samples for the divider supply voltage, yielding an effective output rate of approximately 10 Hz. Since the exact datasheet characteristics for the specific thermistor were unavailable, the nominal Beta coefficient was not utilized to convert resistance to temperature. Instead, an empirical calibration of the complete measurement path was performed against the reference Sensirion SHT35-DIS sensor (Sensirion AG, Stäfa, Switzerland). According to the manufacturer’s specification, the SHT35-DIS operates over a temperature range of −40 to 125 °C, while its typical temperature measurement accuracy is ±0.1 °C within the 20–60 °C range. During calibration, pairs of values consisting of the thermistor resistance and the reference temperature indicated by the SHT35-DIS were recorded. To simplify the computational workflow, a variant of the model representing the relationship between the thermistor resistance and temperature was implemented in the form of a polynomial in x = ln R (R—thermistor resistance), fitted directly to the temperature (rather than its reciprocal) and determined using the least squares regression method over the full set of calibration pairs. Additionally, the variable x was centered relative to its mean value x0 within the calibration dataset, which enhances calculation precision when using single-precision floating-point arithmetic (float32). According to Equation (1) the derived calibration coefficients were determined as follows:

x0 = 11.623290, a0 = 22.641077, a1 = −23.9431, a2 = −0.832025, and a3 = −0.954621.(1)

The validity range of the calibration was restricted to the interval between 16.0 and 55.0 °C. Within this range, the fitting error metrics yielded a root mean square error (RMSE) of 0.0381 °C, a mean absolute error (MAE) of 0.0299 °C, and a maximum absolute error of 0.1421 °C, 3382 calibration data points. These metrics quantify the goodness of fit of the calibration model rather than the absolute measurement accuracy, which is bounded by the reference standard (SHT35-DIS, ±0.1 °C). Treating the reference accuracy and the fit residual as independent contributions yields a combined standard uncertainty u_c_ ≈ 0.12 °C, i.e., an expanded uncertainty U ≈ 0.24 °C (k = 2). All subsequent analyses of the local temperature near the 3D print head were conducted strictly within this validated calibration range.

### 2.2. Linear Dimension Measurements

**Figure 4:**
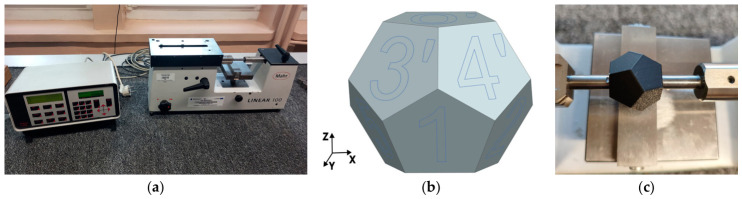
Experimental setup for linear dimension measurements: (**a**) Linear 100 universal length measuring machine used for dimensional accuracy assessment; (**b**) A regular dodecahedron model with numbered faces; (**c**) Close-up of a regular dodecahedron model positioned between the contact tips during measurement under a standardized constant measuring force.

The dimensional accuracy assessment of the manufactured models was conducted using a precise Linear 100 universal length measuring machine (Mahr GmbH, Göttingen, Germany) (Figure 4a). Due to the specific nature of the investigated geometry—a regular dodecahedron—six key linear dimensions were determined for each of the 3D printed objects. These corresponded to the perpendicular distances between pairs of opposite, parallel faces of the solid. To ensure the highest measurement quality, high repeatability, and metrological reliability, the research process was carried out under strictly controlled laboratory conditions (constant ambient temperature of 20 ± 0.5 °C). The applied device featured an indication resolution of 0.001 mm. Prior to the main measurement series, the length measuring machine underwent a calibration procedure using steel gauge blocks. These blocks met the rigorous requirements of the ISO 3650 standard [30], guaranteeing full metrological traceability. To facilitate unambiguous identification and streamline the metrological evaluation, the specific planes of the geometry were systematically designated (Figure 4b). The six faces of the dodecahedron forming its lower half (as viewed from the printer bed) are labeled with numbers from 0 to 5. The faces in the upper half are labeled with numbers from 0 to 5 and a prime symbol. Pairs of parallel faces share the same number in their labeling; for example, faces 4 and 4′ are parallel. Additionally, considering the mechanical properties of the tested PLA material, the measurements were performed applying a standardized, constant measuring force of the contact tips (Figure 4c). This approach allowed for the elimination of systematic errors arising from potential elastic deformations of the polymer surfaces during contact with the measuring elements of the instrument.

### 2.3. Geometrical Measurements

**Figure 5:**
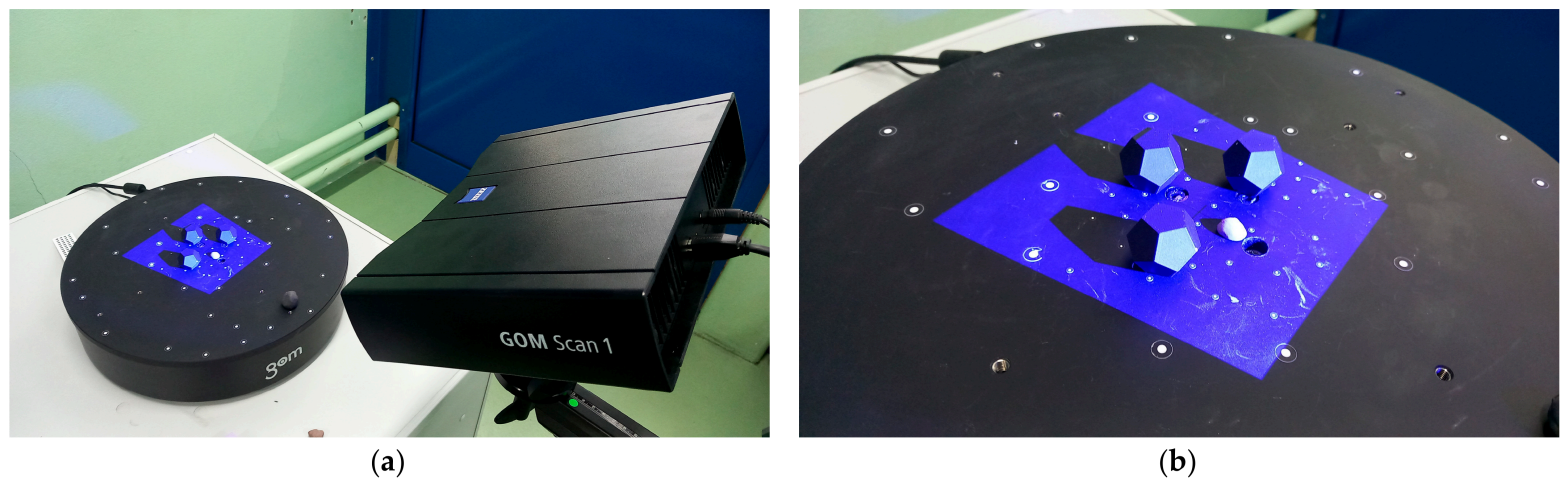
Experimental setup for geometrical measurements: (**a**) GOM Scan 1 optical 3D scanner system equipped with an automated rotary table for 3D digitizing; (**b**) Visualization of geometric measurement.

The methodology for measuring geometric deviations was based on non-contact optical technology utilizing structured light projection. An advanced GOM Scan 1 (version 100) 3D scanner (GOM GmbH (ZEISS), Braunschweig, Germany) utilizing structured blue light was used to digitize the surfaces of the 3D printed models (Figure 5a). 

The scanning head featured a physical sensor resolution of 0.037 mm, a field of view (FOV) of 100 × 65 mm, and a working distance of 400 mm. Given the resolution of the device, if the observed flatness and parallelism deviations are of the same order of magnitude as the scanner resolution, the measurement system may have limited ability to detect differences between specimens. To ensure maximum accuracy, metrological reliability, and measurement traceability, the GOM Scan 1 system was calibrated prior to testing using a certified reference artifact precision dot-matrix calibration plate in accordance with the ISO 10360-8 standard [31]. While the sensor resolution determines the spatial sampling density, the system’s volumetric accuracy was formally verified through a Type B measurement uncertainty evaluation. Based on the calibration certificate and manufacturer specifications, the maximum permissible error (E_MPE_) was accounted for, yielding an expanded measurement uncertainty of U = 0.011 mm (coverage factor k = 2, 95% confidence level). This confirms that the optical measurement system uncertainty (U = 0.011 mm) is substantially smaller than the macro-scale geometric variations (e.g., averaging around 0.05–0.10 mm) evaluated in this study, thereby ensuring adequate measurement capability for flatness and parallelism deviations.

All measurements were conducted under stable environmental conditions within an air-conditioned laboratory, where a standardized reference temperature of 20 ± 0.5 °C was maintained, analogous to the rigorous temperature requirements applied during measurements on a length measuring machine. The measurement procedure was optimized to ensure the highest accuracy and repeatability of data acquisition. Within a single measurement plan, the geometry of three regular dodecahedron models, positioned on an automated rotary table, was digitized simultaneously (Figure 5b). Crucially, the spatial orientation of the models during the scanning process corresponded exactly to their orientation on the 3D printer’s build platform; thus, the surface marked as “0” (the base and adhesion plane) remained in constant contact with the surface of the rotary table. The scanning process involved executing a sequence of 10 automatic table rotations.

The measurement process was conducted in stages by grouping the examined objects. After measuring the first three parts (models 1 to 3), the groups of models designated as 4 to 6 and subsequently 7 to 9 were measured in an analogous manner, utilizing the same automated rotary table parameters and constant model orientation. The aforementioned procedure was comprehensively applied to the selected 3D printing series. The analysis evaluated models fabricated under both investigated 3D printing strategies: the layer-by-layer printing strategy and the model-by-model 3D printing strategy, executed on both tested 3D printers.

### 2.4. Surface Roughness Measurements

**Figure 6:**
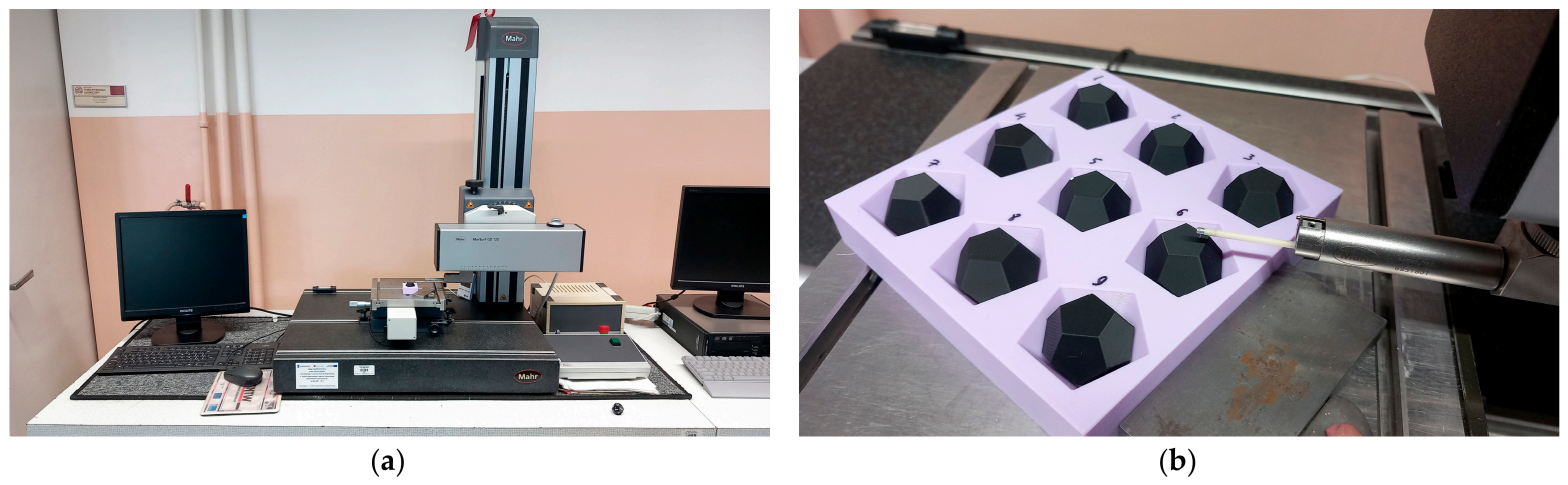
Experimental setup for surface roughness measurement: (**a**) Mahr profilometer equipped with XT 20 analytical software (version 8.00-23 SP 3); (**b**) Contact probe during profile registration on a selected face of the regular dodecahedron model, placed in a stabilizing positioning fixture.

Surface roughness measurements of the 3D printed models were conducted using a MarSurf XR 20 laboratory tactile profilometer (Mahr GmbH, Göttingen, Germany), equipped with a MarSurf GD 120 drive unit and a measuring stylus with a tip radius of 2 µm, tip angle 90°, and a nominal tracing force of approximately 0.7 mN (Figure 6). Measurements were performed after instrument calibration in accordance with the manufacturer’s procedure.

Profile registration and analysis were performed on the individual faces of the regular dodecahedron. One roughness profile was acquired on each analysed surface, with the stylus traversing perpendicular to the deposited extrusion paths. The roughness parameter Ra was determined according to its definition provided in the ISO 21920 standard [32,33,34]. The filtering parameters were selected based on preliminary measurements to ensure an appropriate evaluation of the measured profiles. For the side faces and the top face of the dodecahedron, a cut-off length (sampling length) λc = 0.8 mm and an evaluation length of 4.0 mm were applied. For the surface in contact with the printer build platform, which exhibited considerably higher profile irregularities, a cut-off length λc = 2.5 mm and an evaluation length of 10.0 mm were used. In both cases, the evaluation length consisted of five sampling lengths. During the tests, the models were placed in a dedicated positioning fixture, which ensured repeatable alignment and stabilization of each face relative to the traversing direction of the measuring probe. 

### 2.5. Statistical Analysis

Statistical analysis was performed using R programming language (version 4.5.2). The significance level was set at 0.05 for all statistical tests. Regression analysis was applied to evaluate relationships between the investigated variables. Because of the hierarchical structure of the data and the repeated nature of the measurements, a linear mixed-effects model (LMM) was used instead of a conventional full-factorial model. The fixed effects were printer, printing strategy, measurement orientation, and specimen position on the build platform (build location). Selected two-factor interactions were also included for which there was a technological justification and which could affect dimensional accuracy: Printer × Printing strategy, Printer × Orientation, Printing strategy × Orientation, and Printer × Build location. The Printer × Printing strategy interaction was included because the effect of the printing strategy may differ between printers due to structural and thermal differences. The Printer × Build location interaction was included because the effect of build-platform position may depend on the spatial distribution of temperature and airflow within a given printer. The Printer × Orientation interaction was included because dimensional deviations in individual orientations may differ between printers as a result of differences in calibration and the presence of clearances in the motion system. The Printing strategy × Orientation interaction was included because the printing strategy may alter the thermal history and thereby affect deviations depending on the measurement orientation. Manufacturing series and specimen were included as random effects. The manufacturing-series effect represented variability between independent manufacturing runs, whereas the specimen-level random effect accounted for the correlation among all measurements performed on the same dodecahedron. This model structure made it possible to account for dependencies resulting from the hierarchical organization of the data and to partition variability into between-series, between-specimen, and unexplained residual components. The residual component was not interpreted as measurement or manufacturing repeatability, because it may include measurement error, unmodelled process variability, and other sources of within-observation variation not represented by the fixed and random effects.

Model assumptions were assessed using residual diagnostics. Normality of residuals was evaluated using Q–Q plots, while homogeneity of variance and the absence of systematic residual patterns were assessed using residuals-versus-fitted plots. The diagnostic results indicated that the model assumptions were satisfactorily met for all models, with the exception of the model for Ra, as discussed later in the text.

For flatness deviation and bidirectional parallelism index, linear mixed-effects models were fitted to the log-transformed responses to better satisfy the assumptions of normality and homoscedasticity of residuals. Ra values were also logarithmically transformed to improve residual behaviour. Because some departures from the expected residual distribution remained in this case, the stability of the fixed-effect estimates was assessed using parametric bootstrap confidence intervals based on 1000 simulations.

To determine the relative importance of the individual fixed effects, the change in the marginal coefficient of determination (ΔR^2^) was calculated. For each factor, a reduced model was fitted with the effect under investigation removed, and its explanatory ability was then compared with that of the full model. ΔR^2^ was calculated as the difference between the marginal R^2^ of the full model and the marginal R^2^ of the model with the given effect removed. A higher ΔR^2^ value indicates a greater contribution of that factor to explaining variability in the response variable after accounting for the remaining effects included in the model.

In the graphical presentation of the results, prepared using JMP 12 software (SAS Institute Inc., Cary, NC, USA), the variable on the x-axis was, in most cases, treated as a nominal factor. To improve the readability of the plots, the mean values were connected with a smooth line. This line is not a regression line and is intended solely to facilitate the visual interpretation of the results. Error bars represent ±1 standard error of the mean.

An approximate assessment of measurement repeatability was assessed for the measurements of linear deviation, mean 3D deviation, flatness deviation, bidirectional parallelism index, and the Ra parameter measured using cutoff lengths λc = 0.8 mm and λc = 2.5 mm. For this purpose, each characteristic was measured seven times on a randomly selected specimen (including its removal and repositioning), using a single wall/orientation. Repeatability was quantified using the coefficient of variation, expressed as the ratio of the standard deviation to the arithmetic mean and reported as a percentage. Because the evaluation was conducted using a single specimen and a single surface/orientation, the results should be regarded as an approximate assessment of measurement repeatability rather than a full Gage Repeatability and Reproducibility (GR&R) study covering the entire measurement range.

## 3. Results and Discussion

### 3.1. Linear Deviation

**Figure 7:**
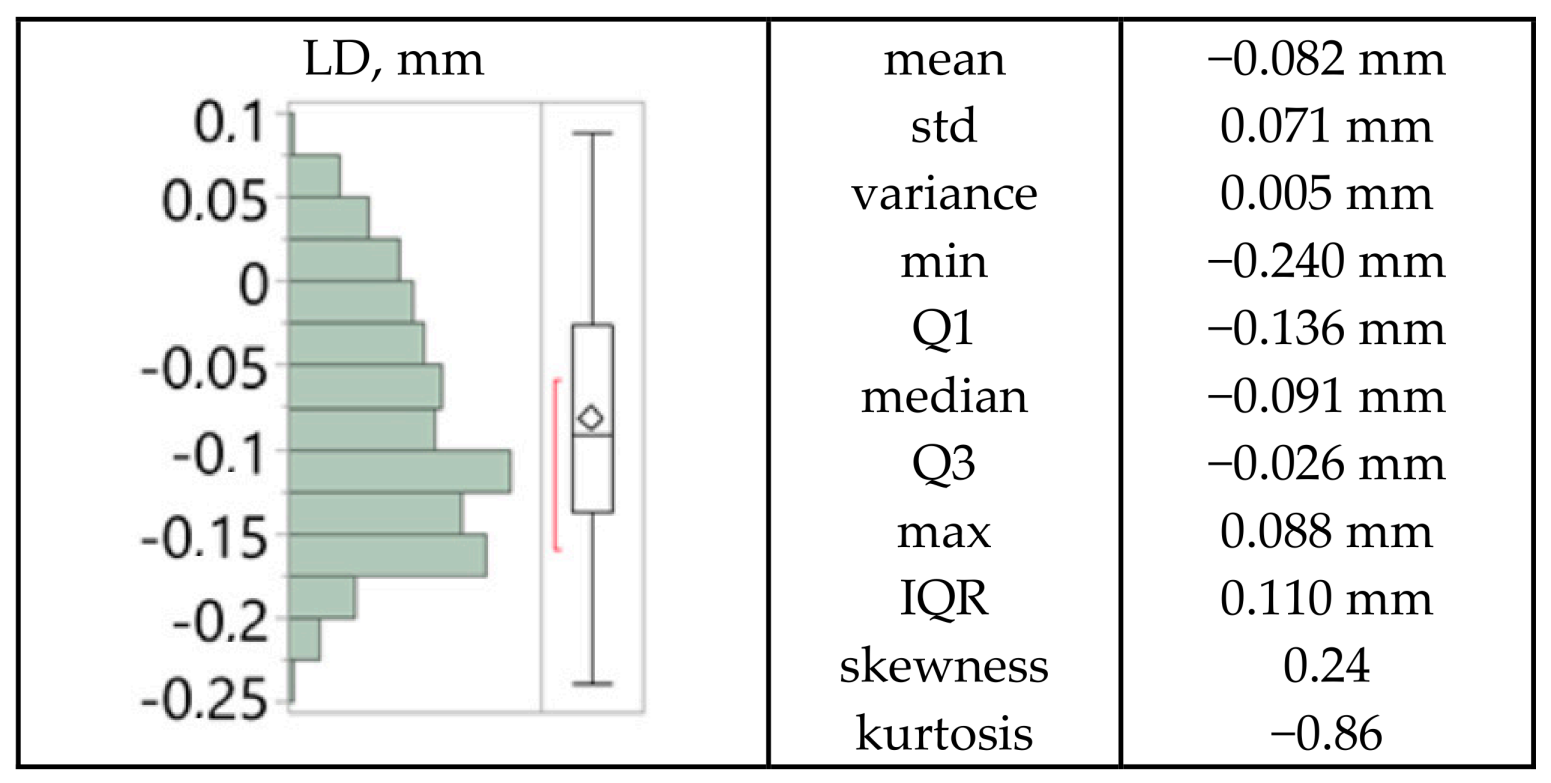
Distribution of LD values and basic descriptive statistics.

The repeatability assessment of the linear dimensional deviation measurements showed low measurement variability, with a coefficient of variation of 0.87%. The vast majority of the deviations (83.56%) were negative. The predominance of negative deviations is associated with the shrinkage of the printed models, which is a typical phenomenon when manufacturing components from thermoplastic materials. The mean deviation value was −0.082 mm. The LD distribution exhibits a slight positive skewness (Figure 7). 

The skewness coefficient is positive but relatively close to zero. The central 50% of the values are located within the range of −0.136 to −0.026 mm, and a slight “tail” is observable at higher (including positive) LD values. Positive deviations were observed exclusively for two pairs of faces: 0-0′ (72% of all positive deviations) and 3-3′ (28% of all positive deviations).

To determine the influence of the investigated 3D printing parameters—printer, build location, and printing strategy—and model-face orientation, a linear mixed-effects model was developed. The fixed-effect tests and relative contributions for linear deviation are presented in Table 3.

**Figure 8:**
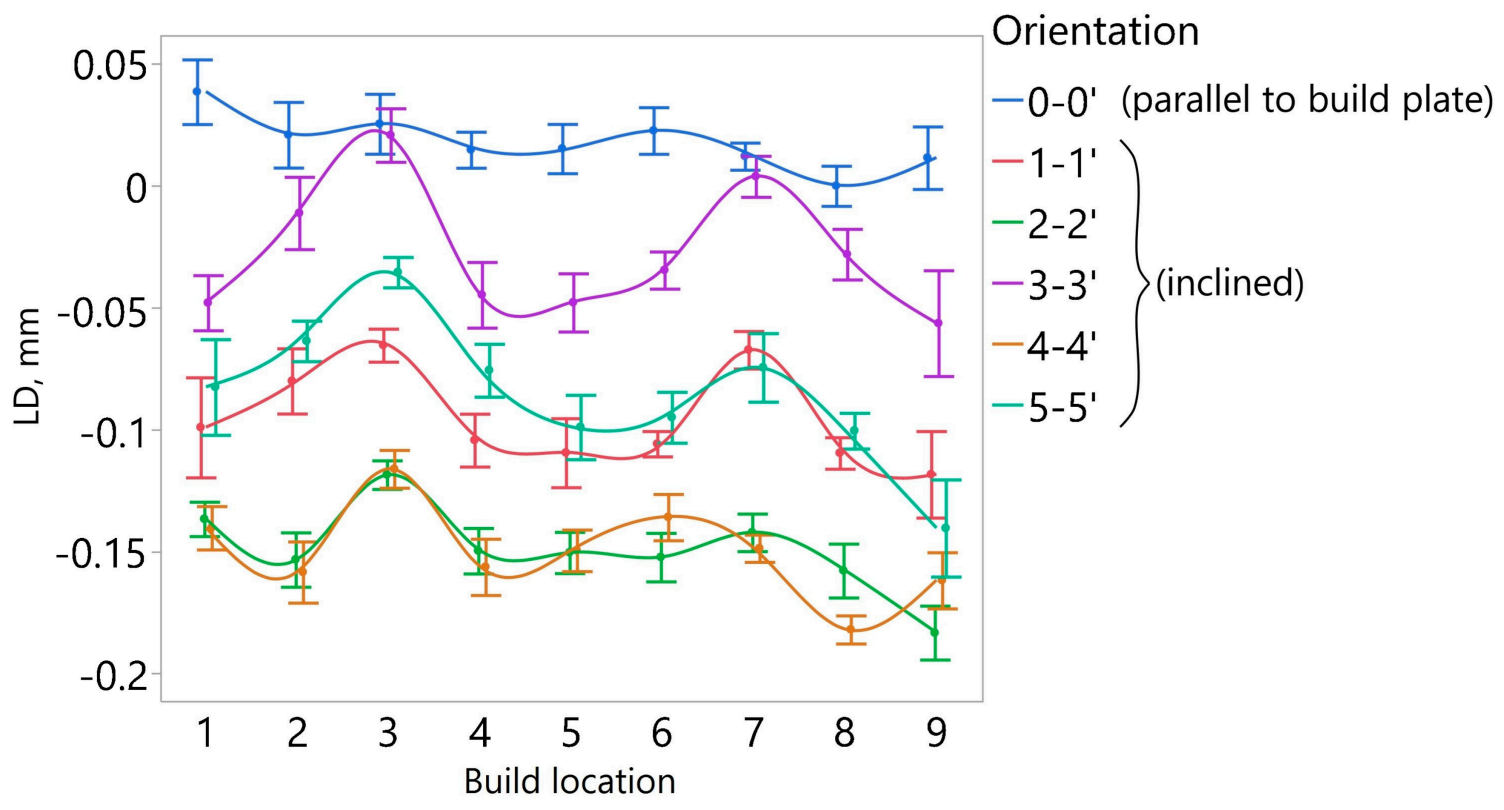
Effect of build location and orientation on LD. Each error bar is constructed using one standard error from the mean. The curves shown are trend lines intended to enhance chart readability.

**Figure 9:**
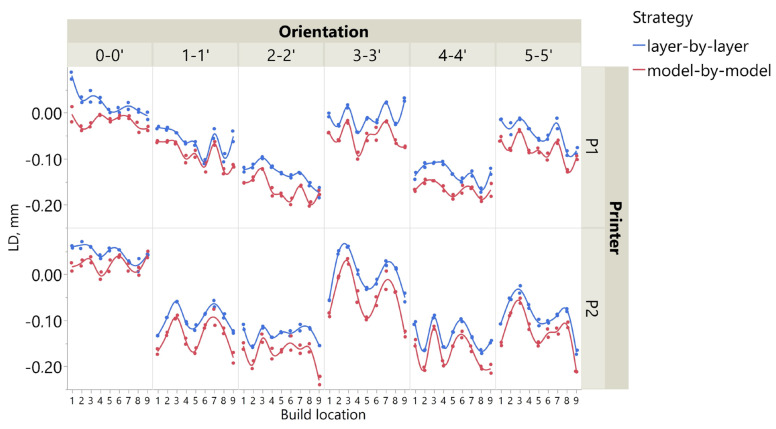
LD value depending on the 3D printing strategy.

**Figure 10:**
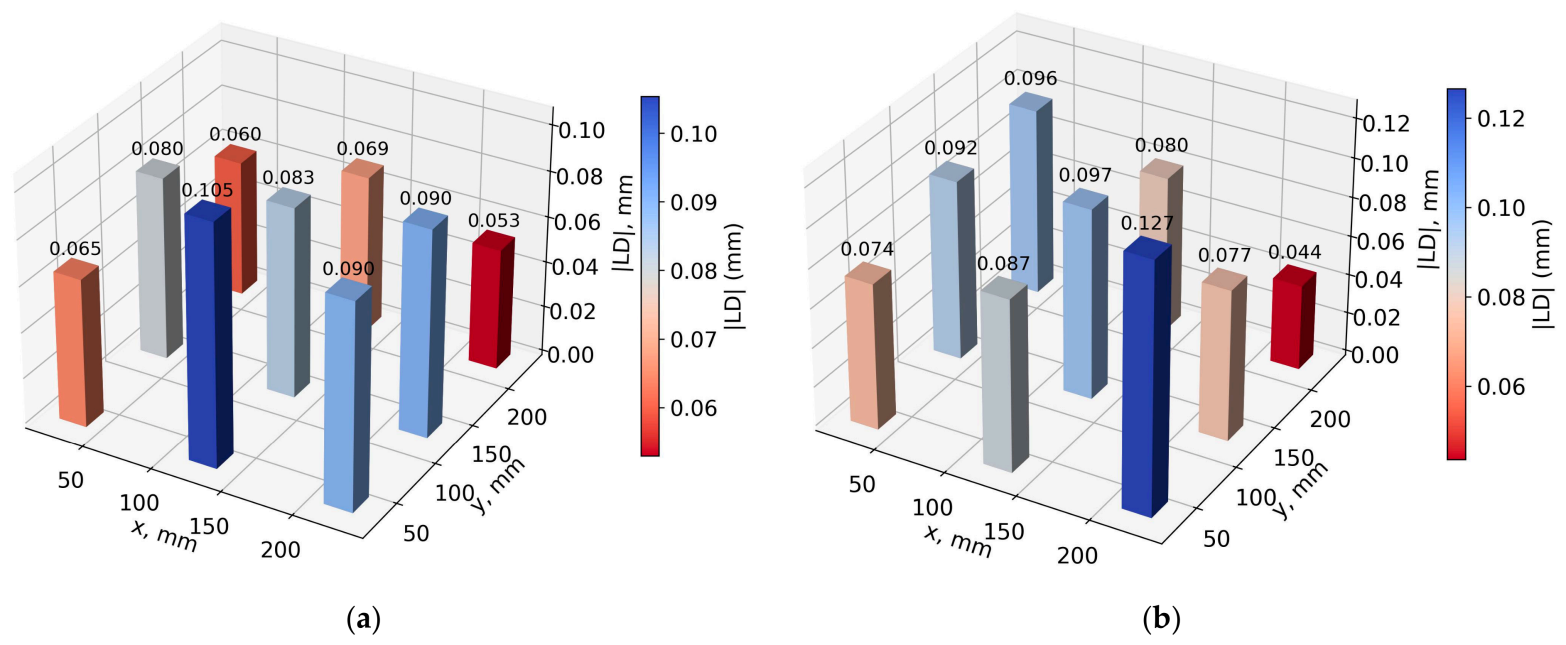
Absolute values of mean LDs for specimens 3D printed in different locations within the workspace of 3D printers: (**a**) P1; (**b**) P2.

**Figure 11:**
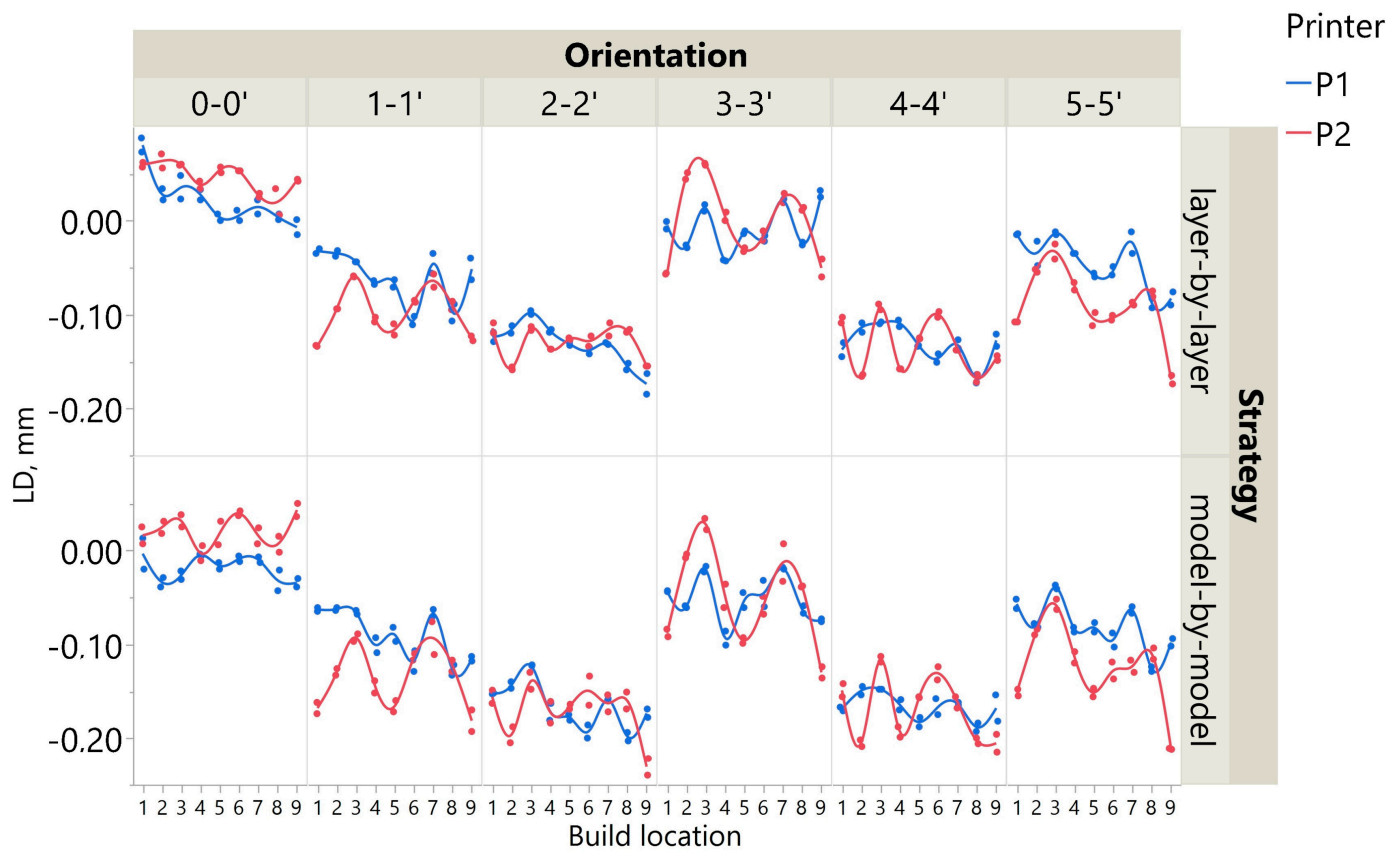
LD value as a function of the 3D printer.

- Face orientation showed the largest relative contribution to explaining variability in linear deviation (ΔR^2^ = 0.776, Figure 8). Different faces are built in varying directions relative to the print axis. In practice, this implies that the part geometry is a far more significant source of variance than the build location within the working space or the 3D printing strategy. The highest LD values were observed for the distances between the faces parallel to the 3D print bed (orientation 0-0′, mean LD value of 0.018 mm), followed by those between faces 3-3′ (mean LD of −0.027 mm). As previously mentioned, a portion of the observed LD values for the 0-0′ and 3-3′ orientations was positive. Lower LD values were observed for the face pairs 5-5′ and 1-1′ (averaging −0.085 mm and −0.095 mm, respectively). The largest negative deviations were recorded for 2-2′ and 4-4′ (averaging −0.149 mm and −0.150 mm, respectively).

- The 3D printing strategy made the second-largest relative contribution to explaining variability in linear deviation (ΔR^2^ = 0.087, Figure 9). These strategies result in distinct heating and cooling profiles within the manufactured components. In the layer-by-layer strategy, all specimens are fabricated concurrently, making the inter-layer interval significantly longer than in the model-by-model strategy. The mean LD value for the layer-by-layer strategy was −0.063 mm. In contrast, the model-by-model strategy exhibited greater shrinkage, resulting in a mean LD of −0.100 mm. As illustrated in Figure 9, the lines connecting the data points for both evaluated strategies are approximately parallel. This indicates a systematic difference in LD across the strategies and points to the absence of significant interactions between the printing strategy and other investigated factors.

- The next largest relative contribution to explaining variability in linear deviation made build location (ΔR^2^ = 0.069, Figure 10). This effect may be related to spatial differences in the thermal conditions within the build chamber. Consequently, the study was extended to include a correlation analysis between temperature and the LD value. The similarity of LD distributions in the build space of two 3D printers (Figure 10) was quantified using the relative root mean square error (RMSE) computed between paired LD values at identical spatial coordinates (x, y), under identical 3D printing strategy, orientation, and corresponding build locations. In addition, Pearson correlation was used to assess the similarity of spatial LD patterns between the two 3D printers. The relative RMSE value was 48%, while the Pearson correlation coefficient reached 0.82. The correlation (r = 0.82) indicates similarity in the spatial pattern of LDs between the 3D printer P1 and P2, whereas the relative RMSE (0.48) reveals substantial differences in the magnitude of these deviations. The possible role of spatial temperature variation was explored further in the subsequent temperature-distribution experiments described in Section 3.4.1.

- The remaining statistically significant effects were the interaction terms, including 3D Printer × Face orientation (ΔR^2^ = 0.037) and 3D Printer × Build location (ΔR^2^ = 0.018). Notably, the main effect of 3D printer does not introduce significant variance, indicating that the mean LD values for both printers were similar. However, the 3D printers differ regarding the spatial distribution of the LD error across the build platform (Figure 10 and Figure 11) as well as the distinct impact of orientation on LD. Each 3D printer may exhibit a unique temperature distribution on the platform, and local thermal conditions (such as heating element arrangement and airflow) can cause the distances between faces in different orientations to vary. Furthermore, the observed variations may stem from minor structural differences (e.g., mechanical backlashes of varying magnitudes or temperature regulation tolerances) and discrepancies in 3D printer calibration.

The marginal and conditional R^2^ values were both 0.913. The random-effect variance estimates for manufacturing series and specimen were at the boundary of zero, indicating that the available data did not support detectable additional variability at these hierarchical levels after accounting for the fixed effects. The residual variability therefore represents variability not explained by the fixed effects included in the model. This residual variance encompasses both the 3D printing process variability and the measurement variability. The measurement variability can be associated with the characteristics of the measuring instrument, such as its resolution and maximum permissible error (MPE), but it also depends on the flatness and parallelism of the surfaces on the evaluated model. The estimated variances for manufacturing series and specimen were at the boundary of zero, indicating that no additional between-series or between-specimen variability was detectable for LD after accounting for the fixed effects.

Face orientation showed the largest relative contribution to explaining variability in linear deviation, followed by printing strategy and build location. Consequently, to enhance dimensional accuracy, optimization efforts should focus on part orientation, 3D printing strategies, and the uniformity of thermal conditions across the build platform. The main effect of the 3D printer was not statistically significant, whereas the interactions involving the 3D printer are pronounced. This implies that both 3D printers are comparable in terms of average accuracy, yet they differ in how they respond to process conditions (namely, orientation and build location on the platform).

The obtained results and the predominance of negative deviations (83.56%) find clear justification in the phenomenon of thermal shrinkage, which is typical for the processing of thermoplastics [18,19]. The observed mean linear deviations of −0.063 mm for the layer-by-layer strategy and −0.100 mm for the model-by-model strategy, measured on a model dimension of approximately 30 mm, correspond to an effective shrinkage of around 0.21% and 0.33%, respectively. These values are within the typical shrinkage range reported for PLA material in the 3D printing process, which generally ranges from 0.2% to 0.5%. The observed differences between the printing strategies may be associated with differences in inter-layer cooling time and thermal history. Reduced inter-layer cooling and greater heat accumulation in the model-by-model strategy constitute plausible explanations for the more negative LD values; however, the thermal history of the specimens was not measured directly in the main experiment.

In the case of the layer-by-layer strategy, there is a significantly longer cooling time between consecutive layers of the same model. Each new filament road is deposited onto a cooler and mechanically stiffer substrate, which partially blocks and constrains the shrinkage of subsequently applied layers, leading to smaller global dimensional deviations. Conversely, in the model-by-model strategy, consecutive layers are deposited much faster, causing the entire model to retain a higher temperature for a longer period, resulting in a larger shrinkage after final cooling, which explains the more negative deviation values. 

This mechanism fits into the broader context of MEX technologies, where process parameters determine the thermal history and directly influence the dimensional errors of the manufactured parts [29,35,36]. Ensuring consistent, repeatable quality remains a challenge due to the sensitivity to variations in material behavior depending on process conditions [37]. Furthermore, the distinct thermal history may lead to a technological trade-off between dimensional stability and surface quality, which is a frequently observed phenomenon when establishing the resolution and speed of additive manufacturing processes [4,38]. The layer-by-layer strategy, while limiting global shrinkage, may result in reduced interlayer remelting and potentially higher surface roughness. On the other hand, rapid deposition in the model-by-model mode promotes better fusion of filament paths, but deteriorates dimensional stability due to greater heat accumulation and subsequent shrinkage. At the same time, face orientation showed the largest relative contribution to explaining variability in linear deviation (ΔR^2^ = 0.776). It confirms that dimensional errors are highly dependent on the complexity of the shape. This also indicates that standard and simplified normative artifacts may be insufficient, and precise verification of the accuracy of processes manufacturing complex shapes requires the use of dedicated, customized measurement geometries [39].

### 3.2. 3D Deviations

The geometrical deviation analysis was performed using specialized Zeiss Inspect metrological software (version 2023). In the first stage of the analysis, the reference CAD model (representing the nominal geometry) and the base point clouds acquired by measuring the physical samples with an advanced GOM Scan 1 optical scanner [40], whose measurement accuracy in structured light technology is crucial for data reliability, were imported into the software environment. Deviation maps were generated for a selected research group consisting of 9 models, manufactured using two distinct 3D printing strategies: layer-by-layer and model-by-model. 

To spatially orient the measurement data relative to the nominal model, a global alignment approach was deliberately chosen to minimize the sensitivity to alignment (registration) errors [40,41]. In additive manufacturing, aligning the scan data to a specific reference plane can severely skew the resulting spatial deviation map due to localized surface irregularities or macro-deformations present on that single plane. The global best-fit strategy evenly distributes the geometric deviations across all faces, making the overall measurement significantly less sensitive to local alignment biases. The choice of this method resulted directly from the adopted measurement strategy. Applying an alignment based on the bottom base (the adhesion surface to the printer’s build platform) would have required changing the part’s clamping in the scanner, performing a second measurement, and subsequently conducting a digital point cloud merging process (stitching). This operation would have introduced an additional, difficult-to-estimate transformation and registration error.

**Figure 12:**
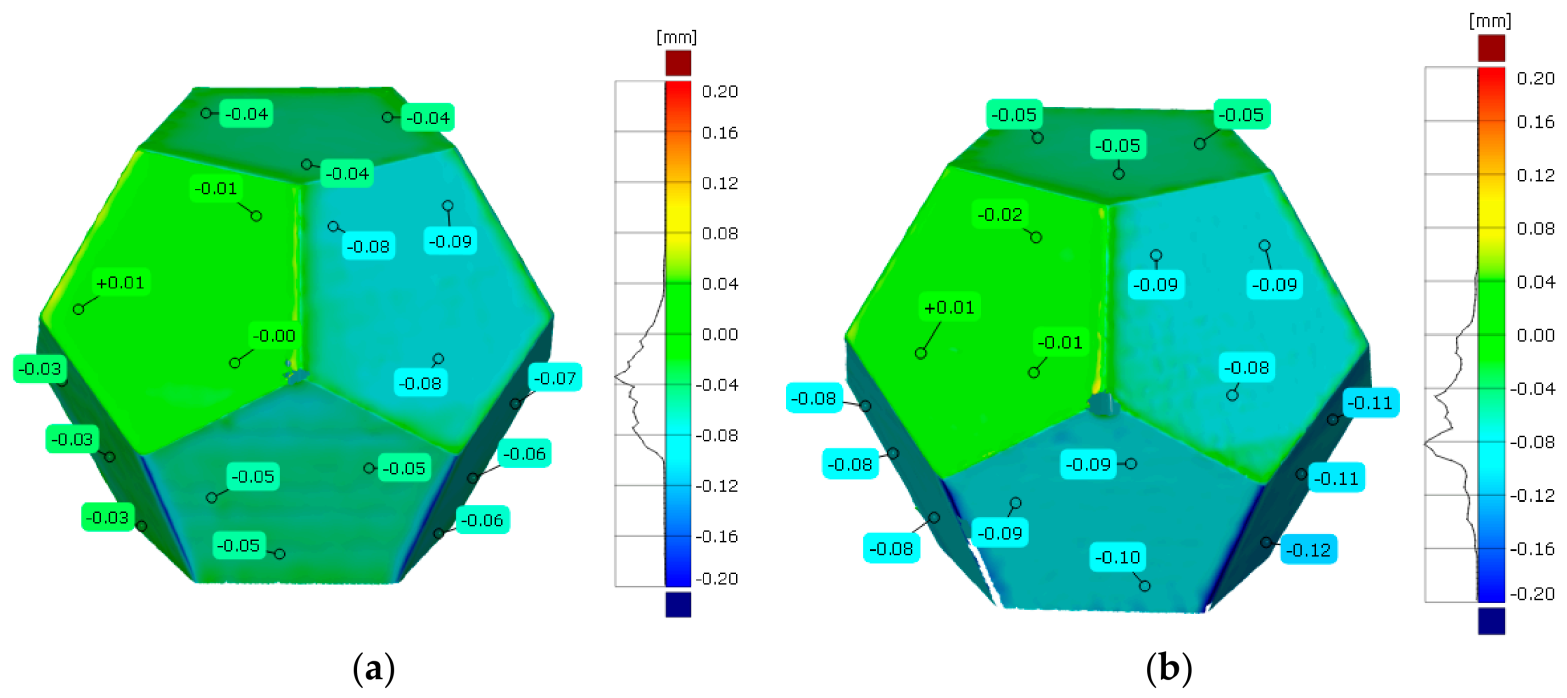
Three-dimensional deviation maps generated for the P1 3D printer in the layer-by-layer strategy for: (**a**) Model 3 (minimum deviations); (**b**) Model 8 (maximum deviations).

**Figure 13:**
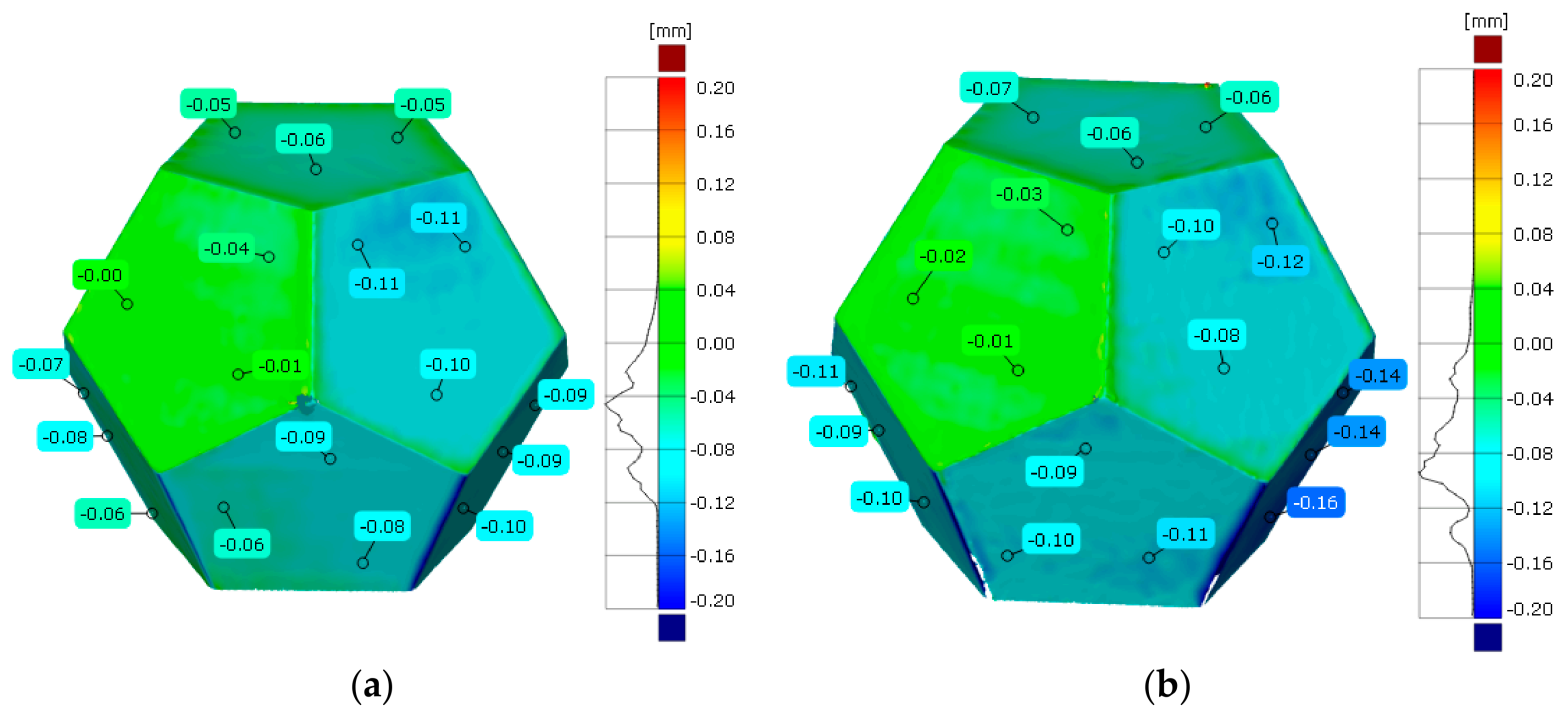
Three-dimensional deviation maps generated for the P1 3D printer in the model-by-model strategy for: (**a**) Model 3 (minimum deviations); (**b**) Model 8 (maximum deviations).

The robustness of this global alignment approach was confirmed during verification, which revealed low average fitting errors: approximately 0.06 mm for the models printed in the model-by-model mode, and slightly lower, around 0.05 mm, for the parts manufactured using the layer-by-layer strategy. After defining the coordinate system through this alignment, the software determined the shortest normal distances between each measurement point of the cloud and the assigned CAD model surface, generating metrological reports in the form of three-dimensional deviation maps. The localization and orientation of the models within the workspace of the P1 3D printer, which resulted in obtaining the maximum and minimum geometric deviations for both analyzed manufacturing strategies (layer-by-layer and model-by-model), are presented in Figure 12 and Figure 13, respectively.

For each sample measured with the scanner, the mean value of all 3D deviations (mean_3D_dev) and the standard 3D deviation (std_3D_dev) were determined. The repeatability assessment of the mean_3D_dev and std_3D_dev measurements yielded coefficients of variation of 3.68% and 2.78%, respectively. The correlation between the mean linear dimension deviation (mean_LD) for a given sample and its mean_3D_dev was examined. Furthermore, the dispersion of LD was compared with that of 3D deviations for each respective sample. A positive correlation was revealed between mean_3D_dev and mean_LD (Pearson’s r = 0.7, *p* < 0.0001). This indicates consistency between both metrics and confirms that the local dimensional errors observed in linear measurements are reflected in the global 3D deviations [35,42]. Considering the values across all analyzed models, the average values of mean_3D_dev and mean_LD were comparable (−0.079 mm and −0.082 mm, respectively). Due to the significantly larger volume of data used to calculate the standard deviation for a given model in the case of 3D deviations, the std_3D_dev values are markedly smaller than those for std_LD [37]. On average, std_3D_dev amounted to 0.048 mm, whereas std_LD reached 0.071mm.

Table 4 presents the fixed-effect tests and relative contributions in the linear mixed-effects model for mean 3D deviation. Build location was the only statistically significant factor, with ΔR^2^ = 0.206. Printing strategy produced a larger change in the marginal coefficient of determination (ΔR^2^ = 0.271), but its *p*-value exceeded the adopted significance level; therefore, this ΔR^2^ estimate should be interpreted with greater caution.

The marginal R^2^ was 0.498, indicating that the fixed effects explained approximately 49.8% of the variability in mean 3D deviation. The conditional R2 increased to 0.721 after including the random effect of manufacturing series, showing that between-series variability made a substantial contribution to the model. Among the random and residual variance components, 44.5% was attributed to differences between manufacturing series, whereas 55.5% remained as residual variability. Residual variability may reflect both unmodelled geometric variation and measurement-related error [35]. The methodology used to determine mean 3D deviation revealed a significant effect of build location and suggested a possible contribution of printing strategy to the observed 3D deviation values [35,37].

### 3.3. Geometrical Deviations of Side Surfaces

The assessment of geometric features concerning flatness and parallelism deviations was performed directly on digitized 3D polygon meshes of the 3D printed dodecahedral samples using ZEISS Inspect software. In the first stage of the metrological procedure, regions of interest (ROIs) were defined for each face of the dodecahedron, from which the corresponding point cloud segments were extracted. Due to the inherent nature of additive manufacturing processes, the surfaces of the 3D-printed specimens are characterized by a distinct macro-structure resulting from layer-by-layer material deposition and visible toolpaths. To address this and separate the high-frequency surface roughness or local waviness—intrinsic to the layered structure—from the actual geometric form errors, the obtained spatial data within the ROIs were subjected to geometric filtration. Specifically, a low-pass Gaussian spatial filter with a cut-off wavelength of λc = 2.5 mm was applied, in accordance with the ISO 16610 standard guidelines [43]. Consequently, this ensured that the evaluated deviations strictly reflected the macro-deformations of the models rather than the local artifacts of the 3D printing process. Based on the filtered data, fitted planes were constructed. To maintain full metrological rigor and avoid the numerical damping of local form errors (typical for Gauss least-squares regression), the Minimum Zone (MZ) method (Chebyshev algorithm) was applied as the fitting criterion for all planes, which is in strict accordance with the ISO 1101 [44] and ISO 12781 standards [45]. The flatness deviation of each face was determined as the minimum distance between two parallel planes enclosing the filtered set of actual points. Similarly, a bidirectional parallelism index was calculated as the minimum width of the tolerance zone bounded by two planes perfectly parallel to the adopted datum. The examination of a bidirectional parallelism index was conducted systematically for five associated pairs of opposite dodecahedral faces, designated respectively as 1–1′, 2–2′, 3–3′, 4–4′, and 5–5′. Because no functional or technological justification existed for selecting one of the opposing faces as a fixed datum, a bidirectional evaluation strategy was adopted. Therefore, the obtained value should be interpreted as a bidirectional parallelism index rather than a conventional single-datum parallelism tolerance according to ISO 1101. In additive manufacturing, surface form errors (such as flatness deviations) vary significantly between individual models and specific faces, making the assignment of a single, rigid datum face for all specimens prone to localized biases. To address this variability and provide a robust metrological assessment, a bidirectional evaluation strategy was implemented for determining parallelism. For each associated pair of opposite faces (e.g., 1 and 1′), the parallelism deviation was measured twice. In the first step, face 1 was established as the primary datum, and face 1′ was defined as the toleranced feature. Subsequently, the layout was reversed, setting face 1′ as the primary datum to evaluate the parallelism of face 1. The final bidirectional parallelism index assigned to a given pair across all models was calculated as the arithmetic mean of these two bidirectional measurements. This averaging approach effectively mitigates the influence of extreme local defects present on any single datum plane, ensuring a highly objective representation of the geometric relationships between opposing surfaces.

Considering the adopted measurement methodology and the limitations associated with the scanner resolution, the variance decomposition results for flatness and parallelism should be interpreted with caution, as they may reflect both process-induced variability and measurement-system limitations.

#### 3.3.1. Flatness

The flatness deviation (FLT) was determined for the faces of the dodecahedron; therefore, instead of focusing on the orientation of parallel faces, each face was considered individually in the study. The average FLT value recorded in the study was 0.055 mm, with a standard deviation of 0.031 mm. This means that the recorded deviations are on the order of the scanner’s resolution. The accuracy of the scanner will therefore significantly influence the determined repeatability, which poses a challenge when assessing geometric accuracy in MEX technology. These difficulties are reflected in the repeatability results. The repeatability assessment of the FLT measurements yielded a coefficient of variation of 11.57%.

Table 5 presents the fixed-effect tests and relative contributions in the linear mixed-effects model for FLT. The marginal and conditional R^2^ values were 0.231 and 0.407, respectively, indicating that the fixed effects explained 23.1% of the variability in flatness deviation, while the inclusion of the random effects increased the explained variability to 40.7%. Of the random and residual variance, 18.5% was attributable to differences between manufacturing series, 4.4% to differences between specimens, and 77.1% remained as residual variability. This high residual component indicates that most of the variability was not captured by the model and may reflect both measurement-related uncertainty and other unmodelled sources of variation [39,46].

**Figure 14:**
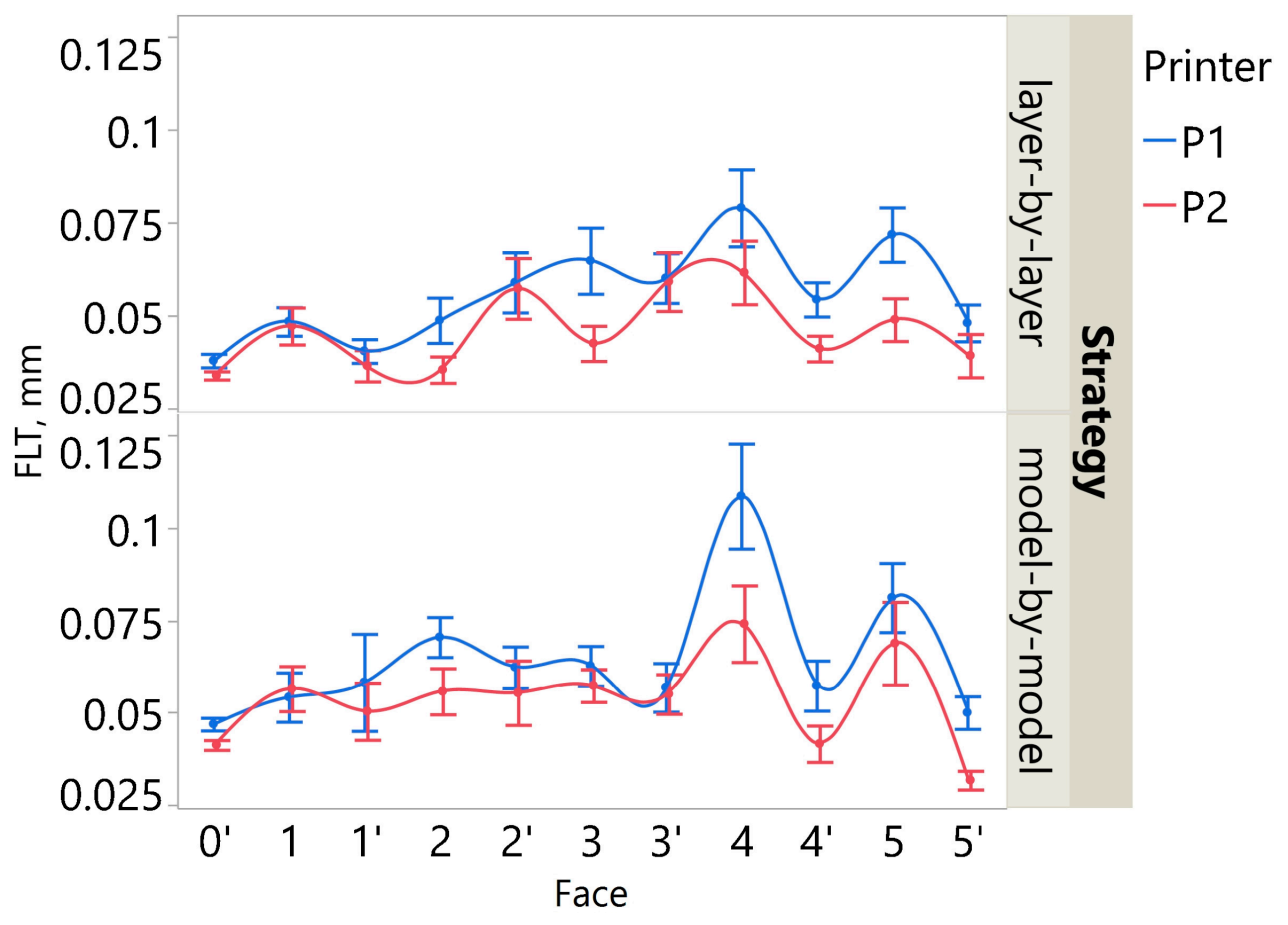
The impact of the printer and wall type on FLT.

**Figure 15:**
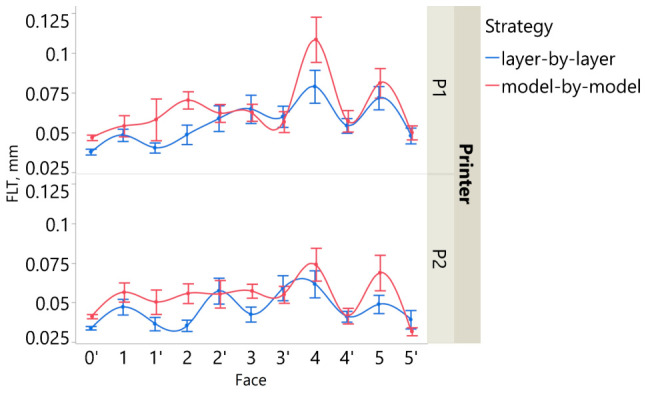
The impact of the 3D printing strategy and wall type on FLT.

Despite the limitations of the FLT measurement methodology, the results revealed a statistically significant effect of wall type, with ΔR^2^ = 0.173, as well as significant interactions of face with printer and printing strategy. However, the corresponding ΔR^2^ values for these interactions were relatively small, at 0.017 and 0.023, respectively. The lowest deviation values were associated with surfaces 3D printed parallel to the build plate at 0°, averaging 0.040 mm, followed by 5° (0.042 mm). The highest FLT values were recorded for walls 4 and 5, at 0.081 mm and 0.068 mm, respectively. As can be observed in Figure 14 and Figure 15, among pairs of parallel walls, those marked with a prime (the upper walls of the model) typically exhibit lower FLT values. The average deviation for the lower walls (without a prime) was 0.062 mm, while for the upper walls (with a prime) it was 0.051 mm. The lower flatness deviation values for the prime-marked walls might be associated with by their formation method during the 3D printing process. A possible explanation is that these walls were built as surfaces tapering upward, ensuring that each subsequent layer was fully supported by the previous one. Conversely, the lower walls represented surfaces expanding upward, which might contribute to the gradual formation of overhangs. Poor support for subsequent layers could potentially facilitate local material deformations, which could lead to higher flatness deviation values [47]. The observed differences may also be partly explained by the stair-stepping effect occurring on inclined surfaces, a phenomenon widely described in the literature as a key factor limiting surface quality in PLA printing [37].

The average FLT value for both printers was 0.060 mm and 0.050 mm, respectively, which means these values differed by 20% (Figure 14). Similar to the case of linear dimensions, the layer-by-layer strategy was associated with lower average FLT values, with an average FLT deviation of 0.051 mm, while the model-by-model strategy resulted in 0.059 mm. As with linear dimensions, this could be hypothesized as an effect of potentially the higher thermal stresses occurring during model-by-model 3D printing [44]. This observation is consistent with the hypothesis that higher thermal stresses associated with temperature variations and inter-layer cooling during model-by-model printing may influence the dimensional stability of PLA components [48,49]. This stability is further modified by the high-dynamic process parameters, which have been reported to influence the final dimensional quality of the manufactured components [35,37].

#### 3.3.2. Parallelism

The bidirectional parallelism index (PAR) incorporates the flatness deviation as well as additional degrees of freedom constraints related to rotation, which are imposed by the established measurement datum. The repeatability assessment of PAR yielded a coefficient of variation of 5.19%. As with FLT, the observed variability may partly reflect limitations of the measurement methodology, which remain a common challenge in additive manufacturing metrology. The average PAR value recorded in the study was 0.097 mm, with a standard deviation of 0.052 mm [50]. 

Two factors were statistically significant: printing strategy and build location, although their associated ΔR^2^ values were relatively small, at 0.085 and 0.060, respectively (Table 6). The marginal and conditional R^2^ values were 0.180 and 0.325 respectively, indicating that the fixed effects explained only 18% of the variability in bidirectional parallelism index, while the inclusion of the random effects increased the explained variability to 32.5%. Of the random and residual variance, only 2.6% was attributable to differences between manufacturing series, 15.1% to differences between specimens, and 82.3% remained as residual variability. This high residual component indicates that most of the variability was not captured by the model and may reflect both measurement-related uncertainty and other unmodelled sources of variation.

**Figure 16:**
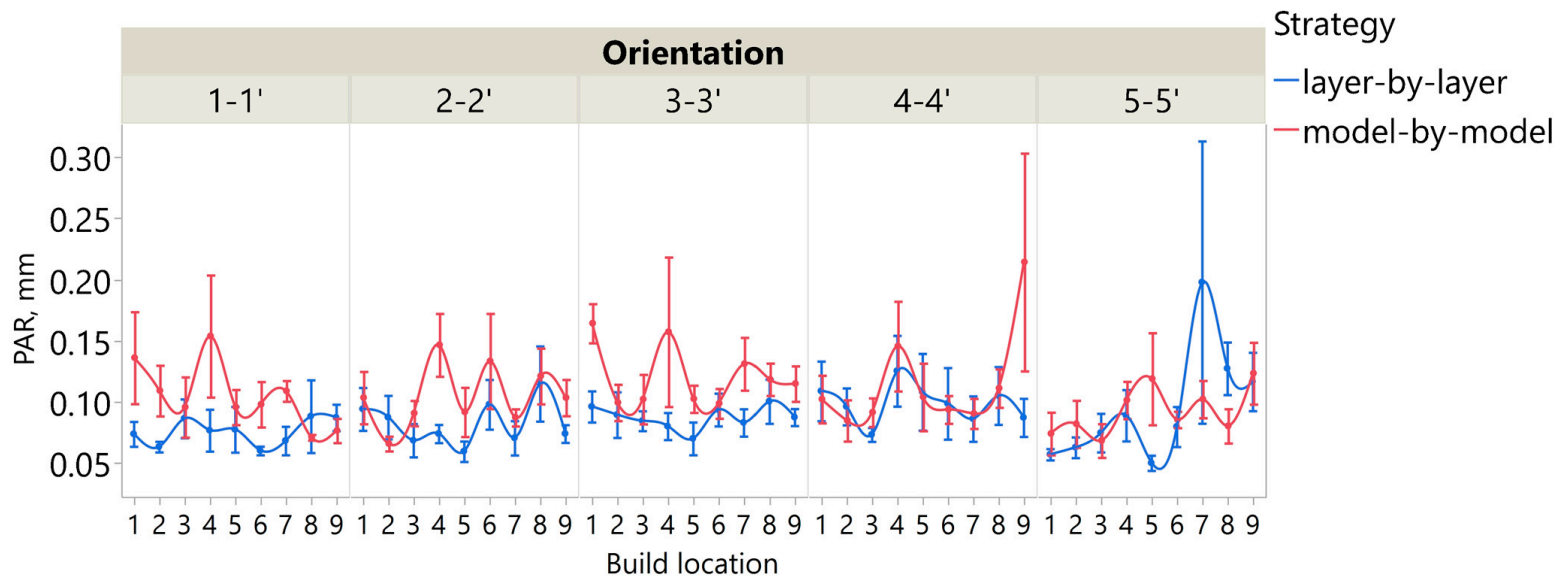
The influence of the 3D printing strategy on PAR.

The layer-by-layer strategy proved to be more favorable, with an average PAR deviation of 0.087 mm, while the model-by-model strategy resulted in 0.107 mm (Figure 16) [50]. However, given the substantial residual variability and the contribution of measurement uncertainty, this difference should be interpreted with caution. The higher deviation in the model-by-model strategy might be related to accumulated thermal stresses, which are hypothesized to have a greater impact on the dimensional stability of the object in this 3D printing mode [47]. These observations suggest that process dynamics and interactions between printing parameters and specimen geometry may contribute to dimensional accuracy, although further studies using higher-precision measurement methods are needed to confirm this interpretation [35].

**Figure 17:**
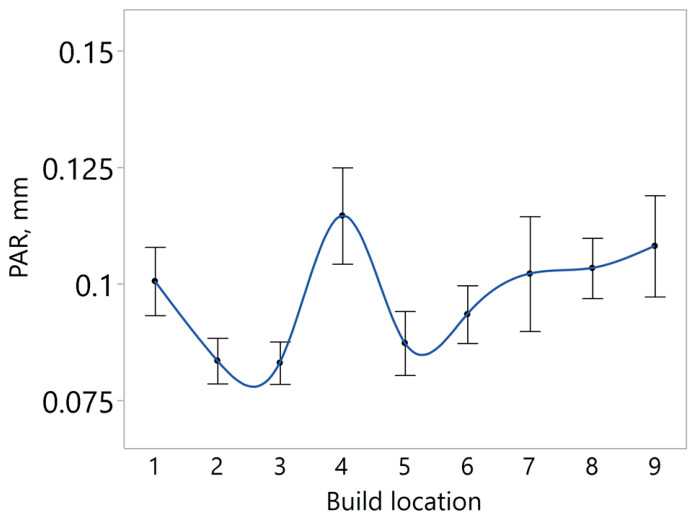
The influence of the 3D printer and build location on PAR.

Figure 17 illustrates the variation in PAR values depending on the build location. The results suggest a possible association between MEX process stability and local thermal and dynamic conditions across the build plate. This is consistent with observations regarding the influence of process parameters on the final quality of the prints [35,37]. However, this interpretation should be treated with caution because a large proportion of the variability remained unexplained and may be attributable to measurement uncertainty.

### 3.4. Additional Studies Evaluating the Effect of Temperature and Seam Location

Additional studies evaluating the effect of temperature and seam location were conducted to systematically address the core optimization challenges in MEX process [46]. These specific parameters were chosen because printing temperature directly controls the thermal gradient and material viscosity, which are the main drivers of volumetric shrinkage [48,49]. Concurrently, the seam location introduces localized material build-up and stress concentration along the deposition paths, frequently resulting in anisotropic dimensional inaccuracies [35,42]. By examining these interrelated factors, this research aims to minimize unexpected geometric deviations and establish reliable tolerance windows for functional PLA components [50].

#### 3.4.1. Temperature

**Figure 18:**
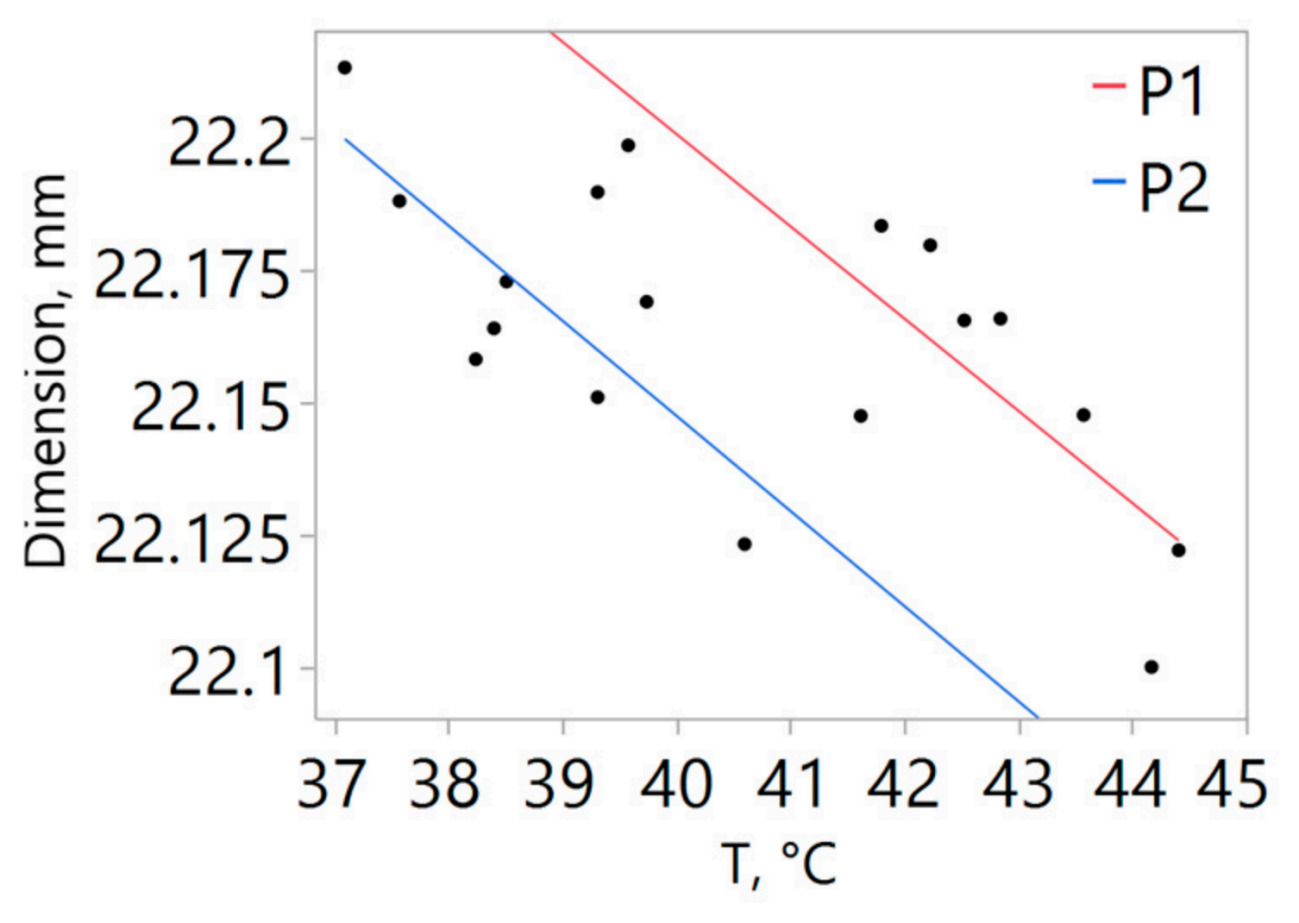
Association between measured temperature and mean linear dimension.

During the research on the influence of various factors on linear deviation (LD), it was observed that model dimensions vary depending on their position within the 3D printer build volume. To investigate whether this is related to the temperature distribution within the workspace, temperatures were measured in the vicinity of the 3D printing zone. Models were 3D printed on two different 3D printers, with each series consisting of 9 models produced in a model-by-model strategy. For each build location, the average temperature during the 3D printing process and the mean linear dimension (averaged from 6 values per model) were determined. Although the temperature sensor placement was similar for both 3D printers, potential differences in positioning precluded a direct comparison of the absolute temperature values. Consequently, a regression analysis was employed to examine the influence of temperature on linear dimension. The regression model accounted for the main effects of temperature and 3D printer, as well as their interaction effect (Table 7). This model is graphically represented in Figure 18.

Assuming a significance level of 0.05, it was demonstrated that the interaction effect between temperature and 3D printer type was not statistically significant (*p* = 0.94). Differences between the printers were revealed; however, as previously mentioned, this is an expected outcome of the differing measurement sensor placements within their chambers. Within the investigated temperature range and experimental conditions, a negative association was observed between the temperature near the printing zone and the mean linear dimension: probably because a higher temperature in the vicinity of the printing zone is associated with greater material shrinkage. This phenomenon stems directly from the thermophysical properties of polymers and the cooling kinetics of the MEX process. A possible explanation is that a higher ambient temperature around the deposited material prolongs the cooling phase. This gives the polymer chains more time to rearrange, which promotes a more intense relaxation of the residual stresses introduced during extrusion, macroscopically leading to deeper volumetric shrinkage [42,49]. Furthermore, exposure of the printing zone to higher temperatures may involve a greater overall thermal gradient (ΔT) upon final cooling of the model to room temperature, geometrically forcing stronger dimensional contraction characteristic of PLA-based materials [48]. The coefficient of determination for the applied model was 0.66 within this additional dataset. This phenomenon may be related to the thermophysical properties of the extruded material. This is hypothesized to give the polymer chains more time to rearrange, which may promote a more intense relaxation of the residual stresses, macroscopically leading to potentially deeper volumetric shrinkage and theoretically forcing stronger dimensional contraction. This association provides indirect evidence of a thermal contribution but does not establish direct causality. Despite the statistically significant association observed in this supplementary dataset, a critical methodological limitation regarding the temperature monitoring configuration must be acknowledged. The NTC thermistor was mounted directly on the moving print head, measuring the local ambient temperature proximal to the nozzle rather than the actual, time-resolved thermal history experienced by the models themselves or the precise temperature distribution across the build plate surface. Furthermore, to ensure clarity in the statistical formulation, the dependent variable of the regression model was defined as the mean linear dimension (averaged across the 6 face-to-face measurements for each specimen), with temperature and printer ID as independent predictors. While this print-head-mounted approach effectively captures macro-level trends in environmental temperature fluctuations within the build chamber, it represents an indirect proxy for the actual thermal exposure of the parts at distinct spatial locations. This measurement constraint introduces a degree of uncertainty into the regression analysis between temperature and linear deviation. Therefore, the current analysis should be interpreted as providing indirect evidence of thermal influence rather than definitive proof of direct physical causality. To obtain a more complete and direct characterization of the thermal field in future investigations, advanced in situ monitoring techniques should be employed, such as multi-point temperature monitoring (e.g., embedding thermocouples directly into the build plate) or high-resolution infrared (IR) thermography. This would allow for a precise decoupling of convection currents, nozzle-proximate radiation, and the true thermodynamic state of the printed parts.

#### 3.4.2. Exploratory Assessment of Seam Configuration

**Figure 19:**
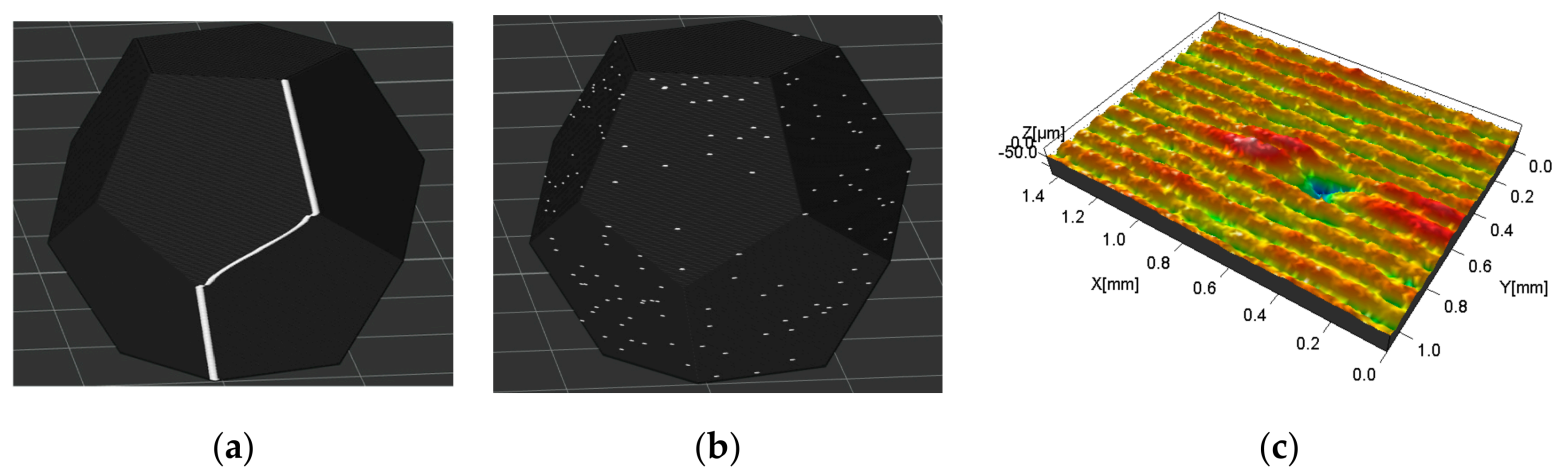
Seam placement: (**a**) aligned; (**b**) random; (**c**) a view of a seam fragment on the topography map in pseudocolors.

**Figure 20:**
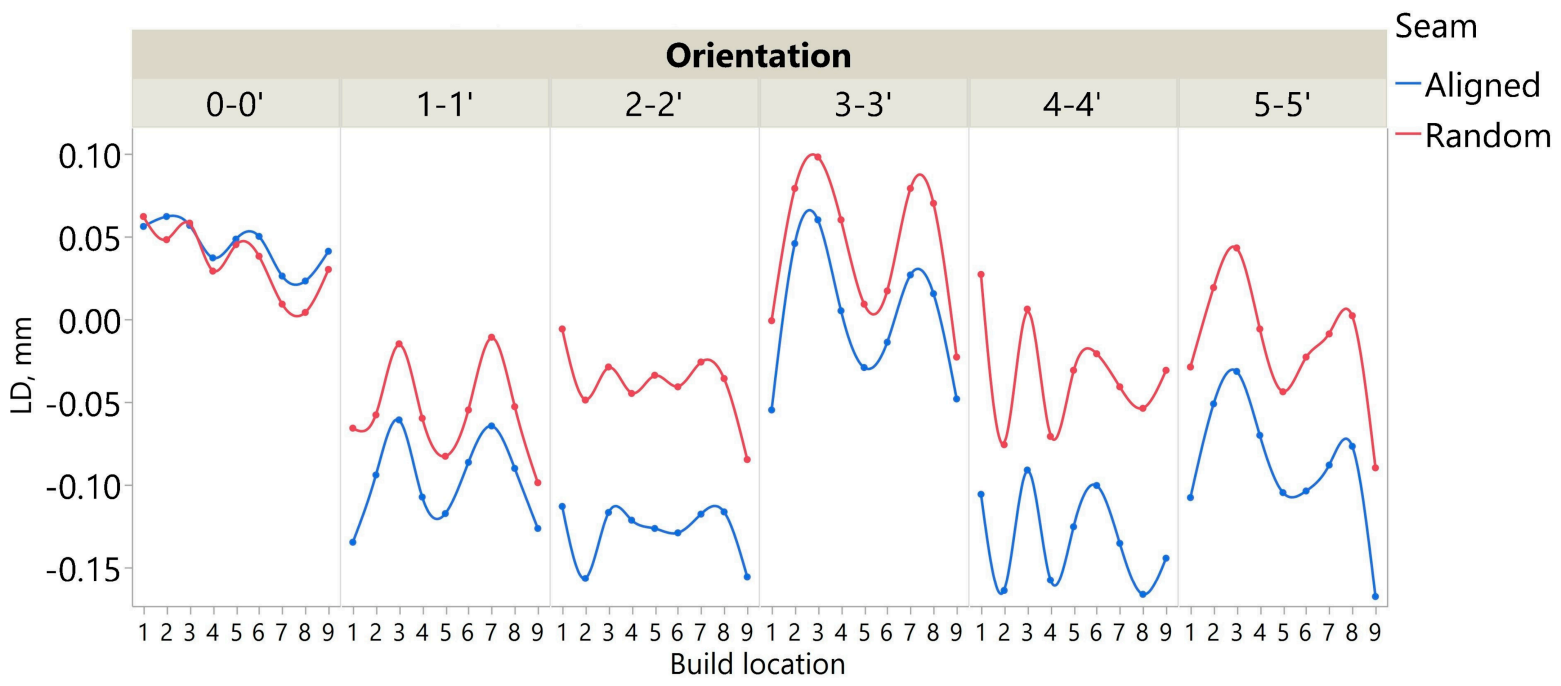
Descriptive comparison of LD values between the aligned and random seam configurations.

Principal investigations of the LD revealed that the orientation of the walls between which the distance was measured affects the final LD value. To explore whether this phenomenon could be related to the position of the seam defined as aligned (which could induce an asymmetric stress distribution within the model), a series of 9 samples was 3D printed with a random seam using a layer-by-layer strategy. Figure 19 illustrates the seam positioning for both the aligned and random variants. A comparison of the LD values for both seam placement variants is presented in Figure 20. Because this supplementary comparison involved one printer and one independent manufacturing series, the results should be interpreted as exploratory rather than as a fully replicated assessment of the seam effect.

The LD values for orientation 0–0′, which does not directly contain a seam, were similar for both seam configurations. For the side-wall orientations, the general orientation-dependent pattern was comparable between the aligned and random seam configurations. However, the random seam was descriptively associated with LD values approximately 0.07 mm larger on average. These results suggest that seam configuration did not explain the orientation-dependent LD pattern under the investigated conditions. Any potential effect of seam configuration on absolute dimensional values requires further investigation. One plausible explanation involves extrusion dynamics: the start and end points of each toolpath (the seam) are associated with abrupt changes in nozzle pressure, leading to local material over-extrusion. In the random option, these local imperfections are scattered across the entire lateral surface, increasing its roughness and creating an irregular profile. During tactile measurement using a length indicator, the measuring tip rests on the highest peaks of these irregularities (the “protruding” seam fragments visible in Figure 19c), which may increase the measured external dimension relative to the nominal plane of the wall [35].

The persistence of a similar orientation-dependent LD pattern under both seam configurations indicates that seam placement alone is unlikely to account for the observed dimensional anisotropy. Other factors, including toolpath orientation, machine kinematics, belt tension, or non-uniform cooling, may also contribute to this pattern [42,50]. Because these factors were not independently varied or directly measured in the present study, they should be regarded as plausible hypotheses rather than demonstrated mechanisms. 

### 3.5. Surface Roughness

**Figure 21:**
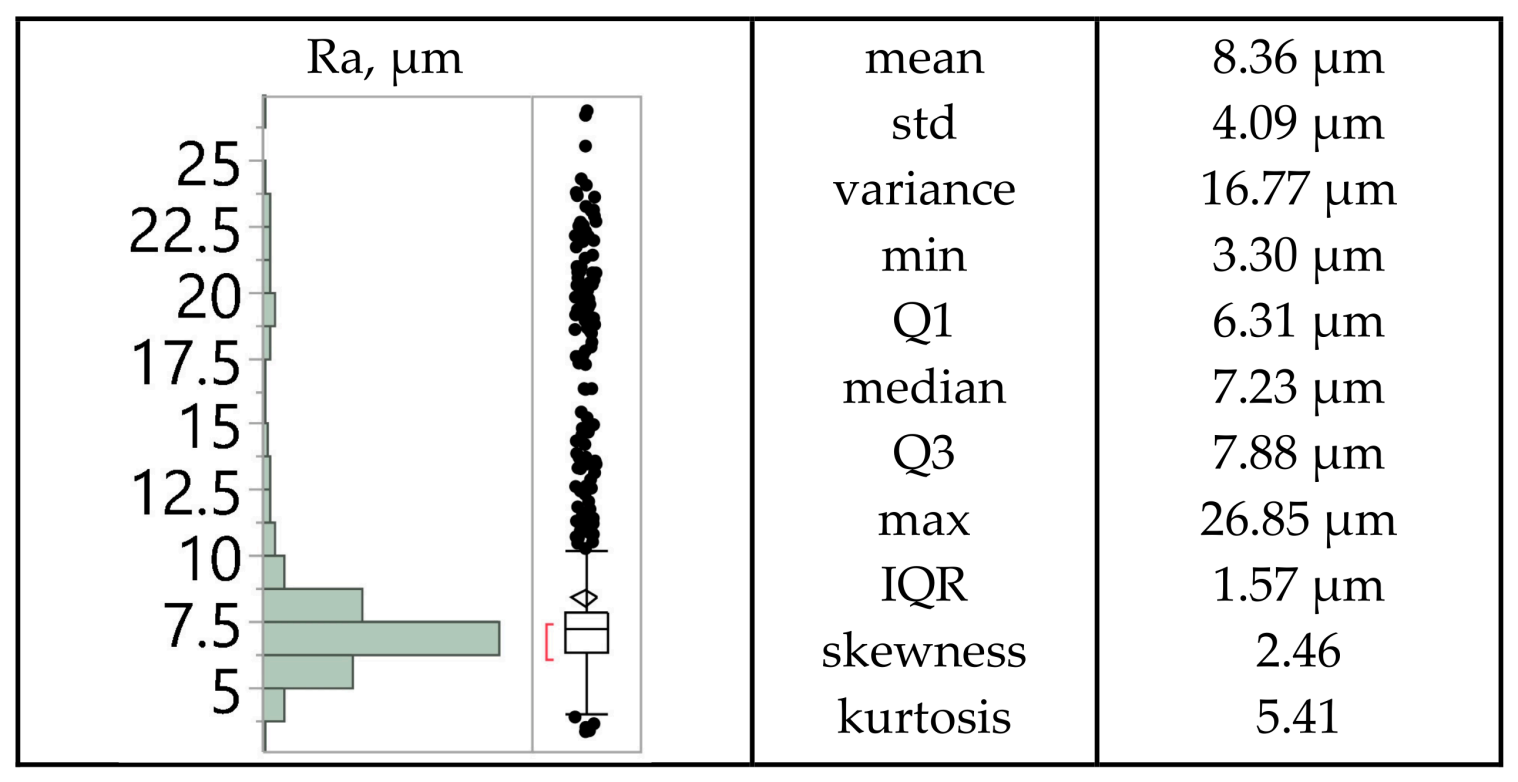
Distribution of Ra values and basic descriptive statistics.

The Ra parameter (arithmetic mean roughness value) was used to evaluate surface roughness. The coefficients of variation for Ra were 3.83% for λc =0.8 mm and 7.77% for λc =2.5 mm. The repeatability of measurements on Face 0, which reproduced the build-platform texture, could potentially be improved by using a longer evaluation length; however, this was not possible in the present study because of the limited specimen size. A single measurement of the Ra parameter was performed on each wall of every sample. The mean value of measured values was 8.36 µm, whereas the median was lower, reaching 7.23 µm, which indicates the presence of isolated high values that skew the mean upward. The distribution of Ra values was characterized by a distinct positive skewness (2.46) and high kurtosis (5.41), demonstrating a concentration of most results near the median, accompanied by the occurrence of outliers (Figure 21). The central 50% of the observations fell within a relatively narrow range from 6.31 to 7.88 µm (IQR = 1.57 µm), while the maximum Ra value reached 26.85 µm, exceeding the median by more than threefold. This implies that the vast majority of the investigated surfaces were characterized by similar roughness, whereas only a few cases exhibited significantly higher Ra values.

**Figure 22:**
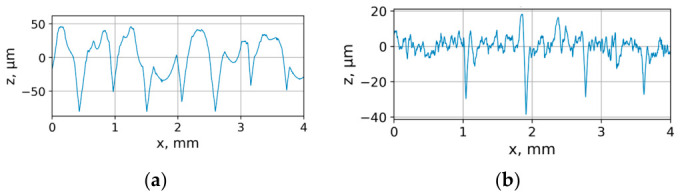
2D roughness profile of the sides parallel to the 3D printer’s XY plane: (**a**) face 0; (**b**) face 0′.

**Figure 23:**
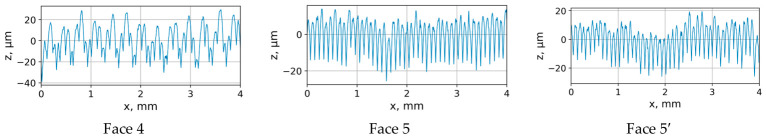
2D surface roughness profile of the model side walls.

The highest Ra values (averaging 20.60 µm) were recorded on face 0, which was in contact with the 3D printer bed; its topography directly reflects that of the 3D printer bed (Figure 22a). The lowest roughness was observed on the top face of the model, which constitutes the final 3D printed layer (average Ra 5.13) (Figure 22b). On the side surfaces of the models, the surface irregularities reflect the successive 3D 3D printed layers (Figure 23). 

**Figure 24:**
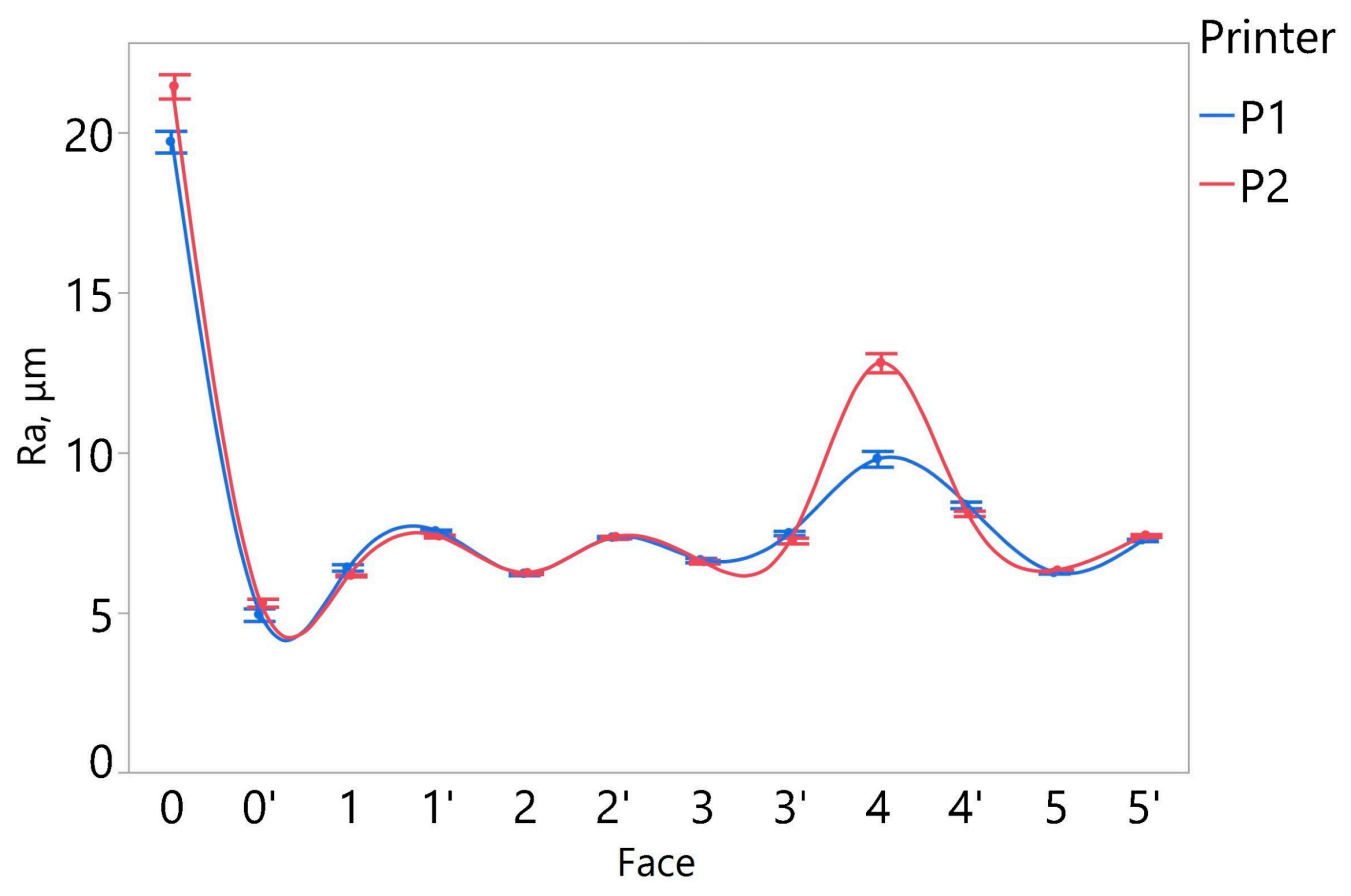
The effect of model wall type and 3D printer on Ra.

Because Surface 0 reflects the texture of the 3D printer bed, its Ra values differ substantially from those of the other surfaces (Figure 24). Therefore, a mixed-effects model was developed for the log-transformed Ra values after excluding Surface 0. The results of this analysis are presented in Table 8. The most influential effect was Face (ΔR^2^ = 0.852), followed by Printer (ΔR^2^ = 0.045). Printing strategy reached statistical significance in the Ra model (*p* = 0.049), but its relative contribution was very small (ΔR^2^ = 0.008) compared with the effect of face identity (ΔR^2^ = 0.852). Its practical influence on Ra may therefore be regarded as limited. The bootstrap confidence intervals generally supported the direction and stability of the principal fixed-effect estimates. Both, the marginal and conditional R^2^ was 0.861, indicating that the fixed effects explained approximately 86% of the variability in Ra. The same values show that the random effect of manufacturing series made a negligible contribution to the model.

Figure 24 demonstrates that among the side surfaces, face 4 stands out due to a noticeably higher roughness compared to the others. Meanwhile, Figure 23 reveals that the layers are shifted relative to one another by up to 40 µm. Analyzing Figure 24 for face 4 also highlights a difference between the 3D printers. Face 4 is the last bottom face to be printed, and the seam is located at its end. In the upper section, the seam is located at the end of face 2′. Figure 24 also shows differences between the bottom faces (1, 2, 3, 5) and their parallel counterparts from the upper half of the model (1′, 2′, 3′, 5′). The faces from the bottom section exhibit lower roughness (averaging 6.36 µm, excluding face 4) compared to 7.56 (for faces 1′–5′).

These specific topographical differences may be attributed to the complex dynamics of the extrusion process. Literature indicates that MEX process parameters exert a direct influence on surface roughness, dimensional errors, and material density [35]. The distinct roughness anomalies observed on specific faces—such as the seam placement on Face 4—along with the discrepancies between individual printers, may stem from machine kinematics [24]. High speeds and accelerations can lead to surface degradation by inducing structural vibrations generated by the movement of the printhead [24]. These vibrations may directly affect the resolution and dimensional accuracy of the fabricated components [24].

Furthermore, achieving reproducible quality and consistency in polymer material extrusion technology remains a significant challenge due to the variability of process parameters and material behavior during fabrication [37]. The pronounced 3D Printer × Face interaction confirms that standard, normative artifacts may be insufficient for a comprehensive evaluation of processes characterized by high dynamics [39]. Consequently, the utilization of complex geometries represents an essential and complementary approach to verifying errors generated by mechanical and thermal factors on non-orthogonal surfaces [39].

## 4. Conclusions

This study provided a comprehensive evaluation of the dimensional accuracy, geometric deviations, and surface quality of PLA components manufactured using high-dynamics MEX technology. The investigated factors included two 3D printers, nine build locations on the working platform, the orientation of the model faces, and two printing strategies (layer-by-layer and model-by-model). The study was further extended to include an analysis of the association between local temperature near the printing zone and dimensional accuracy as well as an exploratory comparison of linear deviation between aligned and random seam configurations. The conducted variance-based analysis enabled identification of the dominant factors associated with dimensional, geometric, and surface variability and provided an assessment of within-condition manufacturing consistency and inter-machine consistency:

- Consistent linear dimensions and surface roughness under the investigated manufacturing conditions. For linear deviation and surface roughness, variability between independent manufacturing series and specimens was low relative to the investigated factors, indicating consistent process performance under constant printing conditions. Because the experimental design did not separate manufacturing from metrological variability, these results should be interpreted as overall process consistency rather than a formal repeatability estimate.
- Part orientation is the dominant source of dimensional variability. Face orientation showed the largest relative contribution to the variability in linear deviation (ΔR^2^ = 0.776), indicating that build direction relative to the print axes has a greater impact on accuracy than machine-specific factors.
- Local temperature near the printing zone temperature was identified as a potential factor associated with location-dependent dimensional deviations. Although the multivariate model accounted for a significant portion of the overall variability within the supplementary temperature dataset, further direct measurements are needed to isolate the thermal contribution from other processing variables.
- 3D printing strategy primarily affects dimensional accuracy, while its contribution to surface roughness is limited. The layer-by-layer strategy resulted in lower shrinkage and better geometric stability, whereas the model-by-model strategy was associated with larger dimensional deviations, potentially due to greater thermal accumulation.
- Machine influence is secondary but interacts with process conditions. Although the average performance of both printers was comparable, significant interactions with build location and orientation were observed, suggesting possible differences in thermal and kinematic behavior.
- Face identity was the dominant fixed effect in the surface roughness model (ΔR^2^ = 0.852, after excluding surface which reproduced the build-platform texture), suggesting that local geometry and overhang conditions had a substantially greater influence than the other investigated factors.
- Measurement methodology strongly influences detected geometric deviations. A significant portion of flatness and bidirectional parallelism index variability may be influenced by the resolution limits of the optical structured-light scanning method used for surface digitization (GOM Scan 1), highlighting the need for caution when interpreting sub-0.05 mm deviations in parts manufactured using MEX technology. Therefore, any relationships inferred for flatness deviation and bidirectional parallelism index below approximately 0.05 mm should be regarded as tentative hypotheses rather than mechanisms conclusively demonstrated by the present dataset.

## Figures and Tables

**Table 1:** Summary of the experimental design.

Study	Printers	Printing Strategy	Independent Manufacturing Series	Specimens Per Series	Total Specimens	Build Positions	Seam Type	Printing Order	Measurements Per Specimen
Main experiment	2	Layer-by-layer	2 per printer	9	36	9 fixed positions	Aligned	Simultaneous printing	6 linear dimensions; 12 flatness; 5 parallelism; 12 Ra measurements; one 3D scan
Main experiment	2	Model-by-model	2 per printer	9	36	9 fixed positions	Aligned	Fixed manufacturing sequence	6 linear dimensions; 12 flatness; 5 parallelism; 12 Ra measurements; one 3D scan
Additional seam study	1	Layer-by-layer	1	9	9	9 fixed positions	Random	Simultaneous printing	6 linear dimensions
Additional temperature study	2	Model-by-model	1 per printer	9	18	9 fixed positions	Aligned	Fixed manufacturing sequence	6 linear dimensions; local temperature recorded continuously

**Table 2:** Key manufacturing and kinematic parameters of the high-speed MEX process based on the 0.08 mm High Quality profile.

Parameter Category	Slicing Parameter	Value/Setting
Profile and Layer Specification	Predefined Print Profile	0.08 mm High Quality
	Layer Height	0.08 mm
	Initial Layer Height	0.12 mm
	Line Width (Outer wall/Inner wall/Infill)	0.42 mm/0.45 mm/0.45 mm
Infill and Shells	Infill Density	100%
	Infill Pattern	Rectilinear
	Wall Loops	2
Thermal Profile	Nozzle Temperature (First Layer/Other Layers)	220 °C/220 °C
	Textured Plate Temperature (First/Other)	35 °C/35 °C
	Cooling Fan Speed (Part Cooling/Auxiliary)	100%/70%
Kinematic, Dynamics and High Speed Parameters	Outer Wall Speed	60 mm/s
	Inner Wall Speed	120 mm/s
	Infill Speed	150 mm/s
	Travel Speed	500 mm/s
	Max Volumetric Flow Rate	21 mm^3^/s
	Acceleration (Normal/Travel)	10,000 mm/s^2^/10,000 mm/s^2^
	Vibration Compensation	Active (Input Shaping)
Experimental Variables	Seam Position	Aligned/Random
	Print Sequence Mode	Layer-by-layer/Model-by-model

**Table 3:** Fixed-effect tests and relative contributions in the linear mixed-effects model for linear deviation.

Effect	Chi-Square	df	*p*-Value	ΔR^2^
Printer	0.51	1	0.475	0
Printing strategy	36.41	1	<0.001	0.087
Orientation	1035.05	5	<0.001	0.776
Build location	123.85	8	<0.001	0.069
Printer:Printing strategy	0.04	1	0.844	0
Printer:Orientation	176.92	5	<0.001	0.037
Printing strategy:Orientation	5.63	5	0.344	0
Printer:Build location	85.05	8	<0.001	0.018

**Table 4:** Fixed-effect tests and relative contributions in the linear mixed-effects model for mean_3D_dev.

Effect	Chi-Square	df	*p*-Value	ΔR^2^
3D Printer	0.92	1	0.336	0
Printing strategy	3.04	1	0.081	0.271
Build location	27.45	8	<0.001	0.206
3D Printer:Printing strategy	0.16	1	0.687	0
3D Printer:Build location	3.17	8	0.923	0

**Table 5:** Fixed-effect tests and relative contributions in the linear mixed-effects model for log(FLT).

Effect	Chi-Square	df	*p*-Value	ΔR^2^
3D Printer	0.02	1	0.892	0.064
Printing strategy	0.9	1	0.344	0.046
Face	64.56	10	<0.001	0.173
Build location	11.4	8	0.180	0.023
3D Printer:Printing strategy	0	1	0.965	0
3D Printer:Face	20.31	10	0.026	0.017
Printing strategy:Face	27.23	10	0.002	0.023
3D Printer:Build location	2.32	8	0.969	0.003

**Table 6:** Fixed-effect tests and relative contributions in the linear mixed-effects model for log(PAR).

Effect	Chi-Square	df	*p*-Value	ΔR^2^
3D Printer	0.5	1	0.480	0.042
Printing strategy	6.4	1	0.011	0.085
Orientation	7.28	4	0.122	0.046
Build location	16.35	8	0.038	0.060
3D Printer:Printing strategy	0.02	1	0.881	0
3D Printer:Orientation	5.24	4	0.264	0.008
Printing strategy: Orientation	5.83	4	0.212	0.002
3D Printer:Build location	7.5	8	0.484	0.020

**Table 7:** Regression model including temperature, 3D printer, and temperature-by-3D printer interactio.

Term	Estimate	Std Error	t Ratio	Prob>|t|
Intercept	22.88001	0.145848	156.88	<0.0001
3D Printer [P1]	0.026732	0.008081	3.31	0.0052
T, °C	−0.01765	0.003633	−4.86	0.0003
(T, °C-40.6407): 3D Printer [P1]	0.000272	0.003633	0.07	0.9414

**Table 8:** Fixed-effect tests and relative contributions in the linear mixed-effects model for log(Ra).

Effect	Chi-Square	df	*p*-Value	ΔR^2^
3D Printer	11.92	1	<0.001	0.045
Printing strategy	3.86	1	0.049	0.008
Face	1377.38	10	<0.001	0.852
Build location	28.69	8	<0.001	0.010
3D Printer:Printing strategy	0.68	1	0.411	0
3D Printer:Face	223.96	10	<0.001	0.04
Printing strategy:Face	26.87	10	0.003	0.005
3D Printer:Build location	13.95	8	0.083	0.002

## Data Availability

The original contributions presented in this study are included in the article. Further inquiries can be directed to the corresponding author.
